# The Proteogenomics of Prostate Cancer Radioresistance

**DOI:** 10.1158/2767-9764.CRC-24-0292

**Published:** 2024-09-19

**Authors:** Roni Haas, Gavin Frame, Shahbaz Khan, Beth K. Neilsen, Boon Hao Hong, Celestia P.X. Yeo, Takafumi N. Yamaguchi, Enya H.W. Ong, Wenyan Zhao, Benjamin Carlin, Eugenia L.L. Yeo, Kah Min Tan, Yuan Zhe Bugh, Chenghao Zhu, Rupert Hugh-White, Julie Livingstone, Dennis J.J. Poon, Pek Lim Chu, Yash Patel, Shu Tao, Vladimir Ignatchenko, Natalie J. Kurganovs, Geoff S. Higgins, Michelle R. Downes, Andrew Loblaw, Danny Vesprini, Amar U. Kishan, Melvin L.K. Chua, Thomas Kislinger, Paul C. Boutros, Stanley K. Liu

**Affiliations:** 1 Department of Human Genetics, University of California, Los Angeles, Los Angeles, California.; 2 Department of Urology, University of California, Los Angeles, Los Angeles, California.; 3 Jonsson Comprehensive Cancer Center, University of California, Los Angeles, Los Angeles, California.; 4 Institute for Precision Health, University of California, Los Angeles, Los Angeles, California.; 5 Department of Medical Biophysics, University of Toronto, Toronto, Canada.; 6 Sunnybrook Research Institute, Sunnybrook Health Sciences Centre, Toronto, Canada.; 7 Princess Margaret Cancer Centre, University Health Network, Toronto, Canada.; 8 Department of Radiation Oncology, University of California, Los Angeles, Los Angeles, California.; 9 Division of Medical Sciences, National Cancer Centre Singapore, Singapore, Singapore.; 10 Department of Oncology, University of Oxford, Oxford, United Kingdom.; 11 Division of Anatomic Pathology, Laboratory Medicine and Molecular Diagnostics, Sunnybrook Health Sciences Centre, Toronto, Canada.; 12 Department of Laboratory Medicine and Pathobiology, University of Toronto, Toronto, Canada.; 13 Department of Radiation Oncology, University of Toronto, Toronto, Canada.; 14 Division of Radiation Oncology, National Cancer Centre Singapore, Singapore, Singapore.; 15 Duke-NUS Medical School, Singapore, Singapore.

## Abstract

Prostate cancer is frequently treated with radiotherapy. Unfortunately, aggressive radioresistant relapses can arise, and the molecular underpinnings of radioresistance are unknown. Modern clinical radiotherapy is evolving to deliver higher doses of radiation in fewer fractions (hypofractionation). We therefore analyzed genomic, transcriptomic, and proteomic data to characterize prostate cancer radioresistance in cells treated with both conventionally fractionated and hypofractionated radiotherapy. Independent of fractionation schedule, resistance to radiotherapy involved massive genomic instability and abrogation of DNA mismatch repair. Specific prostate cancer driver genes were modulated at the RNA and protein levels, with distinct protein subcellular responses to radiotherapy. Conventional fractionation led to a far more aggressive biomolecular response than hypofractionation. Testing preclinical candidates identified in cell lines, we revealed *POLQ* (DNA Polymerase Theta) as a radiosensitizer. *POLQ*-modulated radioresistance in model systems and was predictive of it in large patient cohorts. The molecular response to radiation is highly multimodal and sheds light on prostate cancer lethality.

**Significance:**

Radiation is standard of care in prostate cancer. Yet, we have little understanding of its failure. We demonstrate a new paradigm that radioresistance is fractionation specific and identified *POLQ* as a radioresistance modulator.

## Introduction

Prostate cancer is the second most common cause of cancer death in men (1). It is usually curable when localized, so standard of care is curative intent treatment with either surgery (radical prostatectomy, or RP) or image-guided radiotherapy (RT). Both are equally effective in this setting (2), and decisions are often made based on the side effects and comorbidities. For example, in patients of advanced age, RT is generally preferred (3).

RT involves the delivery of targeted ionizing radiation with the intent of damaging intracellular molecules (4). The common assumption is that DNA is the main molecular target of RT, although proteins and lipids are also ionized (5). RT may also trigger the immune system and modulate the tumor microenvironment. Indeed tumor metastases can be attacked by the immune system after RT as part of a rare phenomenon called the abscopal effect (6). Thus, RT exerts its therapeutic effects through a complex set of molecular and cellular responses.

Classical RT used a limited number of larger treatment fields, which would expose normal cells to significant amounts of radiation, causing dose-limiting toxicities (4). Two main strategies have been taken to reduce radiotoxicities. First, advanced image-guidance is used to precisely target tumors and reduce the dose of radiation that normal cells experience (7). Second, conventional fractionation (CF) schedules deliver the total prescribed radiation dose in multiple smaller daily doses (∼2 Gray, Gy) known as fractions, over several weeks to allow for normal tissue recovery between treatments (8).

As image-guidance has improved (9), it is increasingly viable to deliver radiation to the tumor with minimal normal tissue exposure. This allows the use of higher doses, which might cause more severe cell damage. Hypofractionation (HF), or the delivery of fractions sized 2.5 Gy per day or higher, shortens the treatment time and thereby reduces time for potential tumor cell recovery while increasing patient convenience and compliance. Modern CF and HF have similar rates of toxicity, biochemical failure, metastasis, and overall survival (7, 9–13). Radiorecurrence caused by radioresistance can occur following any fractionation schedule and often results in aggressive disease that is clinically challenging to manage (14–16). The specific molecular and cellular mechanisms underlying radioresistance are unknown, and it is unclear if CF and HF lead to similar or different forms of radioresistance.

To fill this gap in our knowledge, we characterized the proteogenomic response to radiation in both model systems and patient samples. Independent of fractionation, radiation resulted in widespread genomic instability and subsequent gene expression changes in cancer driver genes. HF-resistant cells showed less-aggressive molecular phenotypes than CF-resistant ones. Integration of model system and primary prostate cancer patient molecular data identified *POLQ* (DNA Polymerase Theta) as a candidate radiosensitizer. Genetic or pharmacologic inhibition of *POLQ* resensitized radioresistant prostate cancer cells and induced proteomic signatures detectable in primary prostate cancer. Taken together, these data demonstrate how the precise fractionation schedule applied can significantly change cellular responses to RT. The adoption of clinical hypofractionation may challenge results observed in conventionally fractionated cohorts and model systems.

## Materials and Methods

### Cell culture

Human prostate adenocarcinoma cell line DU145 (RRID:CVCL_0105) was purchased from the American Type Culture Collection (ATCC). DU145-CF (2 Gy × 59 fractions) and DU145-HF (5 Gy × 10 fractions) resistant cells were generated according to previously described methods (17, 18). The process was performed once for each cell type. The characterizations of the DU145 CF and HF cells have been previously reported (17, 18), which include radiation clonogenic, proliferation, invasive capacity, and tumorigenic potential assays. After thawing, DU145-PAR, CF, and HF cells were cultured for 2 weeks prior to experimentation, to allow cell recovery from cryopreservation. We have noted maintenance of the radiation-resistant phenotype of both the CF and HF cells for a minimum of 4 months in culture.

After generation, each cell type was divided into three plates, with each plate cultured independently, resulting in three biological replicates for each sample. Cells were cultured in tissue-culture flasks using Dulbecco’s Modified Eagle Medium containing 4.5 g/L D-glucose and GlutaMAX (DMEM; Gibco) supplemented with 10% fetal bovine serum (FBS) and 1% penicillin-streptomycin. The cells were kept at 37°C in a humidified incubator with 5% CO_2_ and were passaged when they reached 80% confluency.

Parental 22Rv1 prostate carcinoma cell line (RRID:CVCL 1045) was purchased from the American Type Culture Collection (ATCC). An isogenic CF-radioresistant 22Rv1 cell line was created by pooling the surviving cells following a CF irradiation of 2 Gy for 45 fractions on parental 22Rv1. Thawed cells were passaged twice before use. All cell culture reagents were purchased from Gibco. Both cfRR and parental 22Rv1 cell lines were maintained with RPMI-1640 medium supplemented with 10% fetal bovine serum, 100 U/mL penicillin-streptomycin, 1 mmol/L sodium pyruvate, 2 mmol/L L-glutamine, and 1% non-essential amino acids and incubated at 37°C in a humidified atmosphere with 5% CO_2_.

The DU145 and 22Rv1 cell lines were purchased from ATCC which ensures cell line authentication prior to distribution using morphology, karyotyping, polymerase chain reaction (PCR)–based approaches, cytochrome c oxidase I (COI) analysis, and short tandem repeat profiling. CF and HF cells were established directly from these cells. All cell lines tested negative for mycoplasma contamination. MycoAlert PLUS Mycoplasma Detection Kit (Lonza) was used for mycoplasma testing in DU145 cells in July 2019. EZ-PCR Mycoplasma Detection Kit (Biological Industries) following the manufacturer’s protocol was used for mycoplasma testing in 22Rv1 cells on October 15, 2018.

### DU145 DNA and RNA extraction

DNA was extracted in three biological replicates from each sample type, using the Qiagen DNeasy blood and tissue kit (Qiagen). Total ribosomal-depleted RNA was extracted from three biological replicates for CF- and HF-resistant cells, and two for the parental cells, using RNeasy extraction kit (Qiagen).

### DU145 cell line WGS and data preprocessing

DNA samples were sequenced with Illumina HiSeq 2500 using the V4 chemistry to generate paired end reads of 2 × 125 bases. Sequenced reads were tested with FastQC v0.11.8 (RRID:SCR_014583; ref. 19) for quality assurance prior to alignment. DNA reads were then mapped to the human GRCh38 reference genome using BWA-mem2 v2.2.1 (RRID:SCR_022192; ref. 20) and the aligned SAM files were converted to BAM files using SAMtools v1.12 (RRID:SCR_002105; ref. 21). Next, using Picard Tool’s v2.26.10 (RRID:SCR_006525; ref. 22), the resulting BAM files were sorted in coordinate order, duplicates were marked, and the BAM files were indexed. The indexed bam files went through realignment using the Genome Analysis Toolkit (GATK) v3.7.0 (RRID:SCR_001876; ref. 23) and recalibration using GATK v4.1.9 (23) in 18 pairs of parental and radioresistant samples, such that each radioresistant replicate sample was paired to all parental replicates. That resulted in nine pairs per radioresistant sample (e.g., replicate number 1 of CF-resistant cells was run with parental replicate numbers 1, 2, and 3). Separate BAM files were generated for the radioresistant and parental samples and their headers were modified using SAMtools v1.12 (RRID:SCR_002105; ref. 21).

### DU145 cell line somatic mutation calling and downstream analysis

Processed bam files from the previous step were taken for mutation calling in the same pairs described above, so that the called mutations are those gained in a given radioresistant sample compared to the parental (the control, which represents a relatively radiosensitive sample). Single-nucleotide variants (SNV) were called separately using three tools, Mutect2 of GATK v4.2.0.0 (23), Strelka2 v2.9.10 (24), and SomaticSniper v1.0.5.0 (RRID:SCR_005108; ref. 25). To increase reliability, SNVs were declared only for sites that were detected in all three tools, across all tested pairs for a given cell type [i.e., SNV was declared if it was detected 27 times (= 3 algorithms × 9 pairs)] for a given radioresistant sample. The called SNVs were annotated using SnpEff v5.0e (RRID:SCR_005191; ref. 26). Mutational signatures were identified using SigProfilerExtractor v1.14 (RRID:SCR_023121; ref. 27) for each replicate separately. All detected signatures were detected in three replicates for both CF- and HF-resistant cells and their average contributions were calculated for visualization.

Structural variants (SV) were identified using DELLY v0.8.7 (RRID:SCR_004603; ref. 28). For reliability, detected sites were filtered to exclude germline SVs. Annotations were obtained using biomaRt v2.54.0 (RRID:SCR_019214; ref. 29) for the entire SV region in case of deletions, amplifications, and translocations and for the breakpoints in case of inversions.

### DU145 cell line RNA sequencing and analysis

RNA Illumina PCR-free libraries were sequenced on the Illumina HiSeq X to generate paired end reads of 2 × 150 bases and about 90 to 100 Gbases of raw data per sample. Raw sequencing data were tested with FastQC v0.11.8 (RRID:SCR_014583; ref. 19) for quality control and reads were trimmed for low-quality bases accordingly, using fastp v0.20.1 (30). RNA reads were aligned to the GRCh38 GENCODE human release 36 using STAR v2.7.6a (RRID:SCR_004463; ref. 31), and transcript counts were obtained using RSEM v1.3.3 (RRID:SCR_013027; ref. 32). Differential RNA-abundance analysis was carried out using DESeq2 v1.30.1 (RRID:SCR_000154; ref. 33) with the lfcShrink function and a normal shrinkage. To highlight strongly affected genes, an additional stringent test was conducted in the same conditions but setting the lfcThreshold parameter to 0.5. FDR (34) was used to account for multiple testing. To identify cancer-activating transcripts (HCAT), the hallmark cancer gene sets v7.4 were downloaded from GSEA (35), and were processed using mGSZ package v1.0 (36) in R. Gene Ontology enrichment analysis for hallmark cancer pathways was conducted using GSEA (35, 37, 38). Fusion transcripts were identified using STAR-Fusion v1.9.1 (39).

### 22Rv1 DNA extraction and whole-exome sequencing

DNA was extracted from both CF-radioresistant and parental cells in three biological replicates each using the QIAamp DNA Mini Kit (Qiagen). Whole-exome sequencing libraries were prepared using Agilent SureSelect Human All ExonV6 Kit (Agilent Technologies) and 150 bp paired-end sequencing was performed using Novaseq 6000 (Illumina) to depths of 100× per sample (at least 10 GB/sample). Both library preparation and sequencing were carried out by NovogeneAIT Genomics Singapore Pte Ltd.

### 22Rv1 cell line somatic mutation calling and downstream analysis

DNA reads were mapped to the human GRCh38 reference genome using BWA-mem2 v2.2.1 (RRID:SCR_022192; ref. 20) and aligned SAM files were converted to BAM files using SAMtools v1.12 (RRID:SCR_002105; ref. 21). Next, BAM files were sorted by query name with SAMtools v1.15.1 (RRID:SCR_002105) and duplicates were marked with MarkDuplicatesSpark [Picard Tool’s v2.26.10 (22)], resulting in coordinate-sorted and indexed BAM. Indel realignment was done with GATK 3.7.0 (RRID:SCR_001876; ref. 23) and the base quality score recalibration (BQSR) was done with GATK 4.2.4.1 (23). Separate BAM files were generated with BQSR and SAMtools 1.12 (RRID:SCR_002105; ref. 21) was used to reheader appropriately for each sample. Mutect2 v4.2.4.1 with the multisample feature was used to call SNVs in all radioresistant replicates against every parental replicate, such that all three radioresistant replicates were run against every parental replicate separately (overall, three outputs). The intersection of SNV sites from the three outputs generated the final list of SNVs in the radioresistant samples.

### 22Rv1 RNA extraction and processing

Total RNA was extracted from both CF-radioresistant and parental cells in three replicates each, using RNeasy Mini Kit (Qiagen). Stranded RNA-seq libraries were prepared by poly(A) mRNA isolation and NEBNext Ultra II Directional RNA Library Prep Kit for Illumina (New England BioLabs). The 150 bp paired-end sequencing was performed using Novaseq 6000 (Illumina) with at least 50 million reads/sample. Both library preparation and sequencing were carried out by NovogeneAIT Genomics Singapore Pte Ltd. Adapter sequences and low quality base calls were removed from the raw sequence reads using Trim Galore (v0.6.4), a wrapper tool around Cutadapt (v2.10; ref. RRID:SCR_011841; ref. 40). Trimmed reads were then mapped to the GRCh38 reference genome using STAR v2.6.1d (31) with standard settings. Gene quantification was performed with the “–quantMode GeneCounts” option in STAR (RRID:SCR_004463; ref. 31).

### DU145 miRNA data creation and analysis

NanoString duplicate runs were created for CF- and HF-resistant cells and for matched parental duplicate samples corresponding to each radioresistant cell type. NanoString data were normalized and differential abundance analysis was performed as recommended by Bhattacharya and colleagues (41)*.* Targets of miRNAs were identified using the getPredictedTargets function (miRNAtap package v.1.32.0) with the default setting. Briefly, this function allowed identifying miRNA targets relying on five different sources [PicTar (42), DIANA-microT (43), TargetScan (44), miRanda (45), and miRDB (46)], while a minimum of two sources were required for a target to be considered. For each radioresistant cell type (CF or HF), we tested if significantly dysregulated genes (whose transcripts are listed in Supplementary Table S1) were targets of significantly dysregulated miRNAs.

### Subcellular fractionation for shotgun proteomics

The subcellular fractionation was performed as previously described with slight modifications (47). Parental, CF-resistant and HF-resistant DU145 cells were washed three times with phosphate-buffered saline (PBS) and pelleted. The cells were homogenized in lysis buffer containing 50 mmol/L Tris-HCL (pH 7.4), 5 mmol/L MgCl_2_, 0.1% Triton X-100, and protease inhibitors and kept on ice for 10 minutes before being further homogenized with a loose-fitting pestle. Sucrose (250 mmol/L) was then added to the lysates to make an isotonic solution, after which the lysates were centrifuged at 800 × *g* for 15 minutes at 4°C to separate the nuclear fraction. The resulting supernatant was used for fractionating cytosol, mitochondria, and microsomes (mixed membranes). The nuclear pellet was resuspended in 2.5 mL of cushion buffer (2 mol/L sucrose, 50 mmol/L Tris-HCl, 5 mmol/L MgCl_2_, 1 mmol/L dithiothreitol (DTT), and Protease inhibitors – Roche) and overlaid on top of 2 mL of cushion buffer in 5 mL ultracentrifugation tube (Beckman) and centrifuged at 80,000 × *g* for 45 minutes (Beckman SW 55Ti rotor) to isolate the nuclear pellet. The mitochondrial fraction was isolated from the crude lysate by centrifugation at 8,000 × *g* for 15 minutes to retrieve the mitochondrial pellet. The microsomal pellet was isolated by centrifuging the supernatant at 150,000 × *g* for 1 hour. The resulting supernatant was the cytosolic fraction. Nuclear proteins were extracted using a lysis buffer containing HEPES, NaCl, and EDTA, after which the pellet was passed through an 18-gauge needle several times and centrifuged to isolate the soluble nuclear fraction and insoluble pellet. Finally, the organelle pellets (mitochondria, nuclear, and microsome) were lysed in [50% (v/v) 2,2,2,-trifluoroethanol and 50% PBS].

The pellets obtained from the subcellular fractions were lysed by repeated freeze-thaw cycles (five cycles, switching between a dry ice/ethanol bath and 60°C water bath) in a lysis buffer. Samples were sonicated on an ultrasonic block sonicator for five 10 seconds cycles at 10 W per tube (Hielscher VialTweeter) followed by extraction at 60°C for 1 hour. Disulfide bonds were reduced with 5 mmol/L DTT, followed by 30 minutes incubation at 60°C. Free sulfhydryl groups were alkylated by incubating the samples with 25 mmol/L iodoacetamide in the dark for 30 minutes at room temperature. The samples were diluted (1:5) with 100 mmol/L ammonium bicarbonate (pH 8.0) and 2 mmol/L CaCl_2_ was added. Proteins were digested overnight with 2 μg of trypsin/Lys-C enzyme mix (Promega) at 37°C. Peptides were desalted by C18-based solid phase extraction, then dried in a SpeedVac vacuum concentrator. Peptides were solubilized in mass spectrometry-grade water with 0.1% formic acid.

### Sample preparation for proteomic analysis following *POLQ* knockdown

Parental CF- and HF-resistant DU145 cells were transfected with scramble siRNA (control) or siRNA to target *POLQ* (see below siRNA transfection and novobiocin inhibitor treatment) in six-well plates and grown to 80% confluency. After being washed three times with cold PBS, cells were gently scraped from the surface of the well and transferred to 1.5 mL microcentrifuge tubes. Cells were pelleted by centrifugation, the PBS was removed and the pellets were flash-frozen to be stored at −80°C until ready for further processing.

To prepare for shotgun proteomics, cell pellets were lysed by repeated freeze-thaw cycles in lysis buffer (50 mmol/L HEPES pH 8, 1% SDS). Samples were sonicated on a probe-less ultrasonic sonicator for five 10 second cycles at 10 W per tube (Hielscher VialTweeter) to shear genomic DNA. Samples were centrifuged at 18,500 × *g* to pellet cell debris and the supernatant was used for subsequent steps. Disulfide bonds were reduced with 5 mmol/L dithiothreitol, followed by 30 minutes incubation at 60°C. Free sulfhydryl groups were alkylated by incubating samples in 25 mmol/L iodoacetamide in the dark for 30 minutes at room temperature. An additional 5 mmol/L of DTT was added to quench the alkylation reaction and samples were incubated at room temperature for 5 minutes. The magnetic bead-based SP3 protocol (48) was used to capture proteins prior to digestion. Briefly, magnetic beads were added to proteins in a 10:1 (w/w) ratio. Absolute ethanol was added to bring the ethanol concentration to 70%. Samples were shaken at room temperature for 5 minutes at 1,000 rpm, and the supernatant was discarded. The beads were rinsed two times with 80% ethanol and discarded. Proteins were digested in 100 mmol/L ammonium bicarbonate containing 2 μg of trypsin/Lys-C enzyme mix (Promega) at 37°C overnight. Peptides were desalted using C18-based solid phase extraction, then lyophilized in a SpeedVac vacuum concentrator. Peptides were solubilized in mass spectrometry-grade water with 0.1% formic acid. LC/MS-MS data were acquired as previously described (49).

### Mass spectrometry sample processing for proteomic analysis

LC/MS-MS data were acquired as previously described (49) with modifications. Peptides (2 μg) were loaded on a two-column setup using 2 cm Acclaim PepMap 10 column (75 μm, 3 μm, 100 Å) as trap column and a 50 cm EasySpray ES803 column (75 μm, 2 μm, 100 Å; Thermo) coupled to an Easy nLC 1,000 (Thermo) nanoflow liquid chromatography system connected to Q-Executive HF mass spectrometer (Thermo). Peptides were separated by reverse-phase chromatography using a 265 minutes nonlinear chromatographic gradient of 4% to 48% buffer B (0.1% FA in ACN) at a flow rate of 250 mL/minutes. Column temperature was kept at 45°C. Mass spectrometry data were acquired in positive-ion data-dependent mode. Data-dependent MS analyses were run in a positive top-25 mode. MS1-spectra were acquired for a mass range of *m*/*z* 350 to 1,800 at a resolution of 120,000, with automatic gain control (AGC) target of 1 to 106 and 45 ms maximum fill time. The dependent MS-MS spectra were acquired at a resolution of 30,000, with an AGC target of 2 × 10^5^ and 55 ms maximum fill time. The isolation window width was set to 1.4 *m*/*z*, the isolation offset to 0.2 *m*/*z*, and the intensity threshold to 1.8 × 10^3^.

### Proteomic data processing and analysis

Subcellular fractionation raw data were searched in MaxQuant (50) v1.5.8.3 using the UniProt protein sequence database containing human protein sequences from UniProt (RRID:SCR_002380; number of sequences 42,041). *POLQ* siRNA raw data were searched MaxQuant v1.6.3.3 (RRID:SCR_014485) using the UniProt protein sequence database containing human protein sequences from UniProt (complete human proteome; 2019-09). Searches were performed with a maximum of two missed cleavages and carbamidomethylation of cysteine as a fixed modification. Variable modifications were set as oxidation at methionine and acetylation (N-term). The FDR for the target-decoy search was set to 1% for protein, peptide, and site levels. Intensity-based absolute quantification (iBAQ), label-free quantitation (LFQ) was enabled, and match between runs were disabled. The proteinGroups.txt file was used for subsequent analysis. Proteins matching decoy and contaminant sequences were removed and proteins identified with two or more unique peptides were carried forward.

For fractionation data, log_2_-transformed iBAQ intensities were used. If a protein was detected for two out of three replicas for a given sample type, the third value was imputed based on the mean of the two others. All other missing values were imputed from the lower half of the Gaussian distribution (width of 0.3 and downshift of 1.8; ref. 51). Weighted Gene Co-expression Network Analysis (WGCNA) was performed using WGCNA package in R v1.72-1 (RRID:SCR_003302; refs. 52, 53) based on protein abundance in specific subcellular fractions in CF- and HF-resistant cells. Enrichment analysis of module genes was performed using gprofiler2 v0.2.1 (54). Differences between protein abundances were evaluated between the parental cells (control) and each of the radioresistant cell lines in whole cells as well as every subcellular fraction using two-sided *t* tests in R. Three replicates were used for CF- and HF- resistant cells in whole cells or subcellular fractions. For the parental cells, two replicates were used for the microsome, mitochondria, nuclear soluble, and plasma membrane and three replicates otherwise.

For protein quantitation (55) siRNA knockdown experiments, LFQ intensities were used. Missing LFQ values were replaced with median-adjusted iBAQ values (51). Protein intensities were log_2_-transformed for further analysis. Differences between protein abundances were evaluated between *POLQ*-depleted samples and the corresponding control for the parental and CF-resistant cells, using two-sided *t* tests in R. Three biological replicates were used for each sample type. FDR (56) was used to control multiple testing. Pathway enrichment analyses were performed using the gseGO function of the ClusterProfile package in R v4.6.0 (57, 58), relying on gene lists that were pre-ranked based on effect sizes.

### Pearson RNA–protein correlations in DU145 cell lines

Pearson correlations were calculated between median RNA counts and median protein intensities for each cellular fraction, across all genes or cancer hallmark genes detected in both platforms. For this goal, only non-imputed protein intensities were used. Genes for which the RNA or protein median values were zero were excluded.

### siRNA transfection and novobiocin inhibitor treatment

To achieve *POLQ* knockdown using siRNA degradation, parental, CF-resistant, and HF-resistant DU145 cells were seeded in six-well plates, and 24 hours later, cells were transfected with scrambled siRNA (control; CAT. #: SR30004; Origene Inc.) or *POLQ* siRNA (CAT. #: SR323222; Origene Inc.) using siTran 2.0 Transfection Reagent (CAT. #: TT320001; Origene Inc.) and left overnight for at least 24 hours before performing experiments.

To induce POLQ inhibition with novobiocin, parental, CF-resistant, and HF-resistant cells were seeded in six-well plates overnight, then treated with water (control) or 100 µmol/L novobiocin diluted in water (Cat. #S2492; Selleckchem) the following morning. Cells treated with novobiocin were given 24 hours for POLQ inhibition.

### Clonogenic survival assay

Control and treated cells (*POLQ*-siRNA or novobiocin-treated cells) were plated in triplicate on six-well plates and mock-irradiated at 0 Gy or irradiated using two fractions of 4 Gy with a 24-hour interval in between. The mock-irradiated and irradiated plates were left to incubate at 37°C in a humidified incubator with 5% CO_2_. After 10 to 14 days, the cells were stained with crystal violet (Sigma) and the colonies (defined as being >50 cells) were counted. Surviving fraction of each cell line was determined by dividing the plating efficiency of cells given two fractions of 4 Gy by the plating efficiency of cells given 0 Gy.

### Quantitative real-time PCR

To quantify mRNA abundance, total RNA was extracted from cells using RNeasy Mini Kit (Qiagen), which was then converted to cDNA for amplification using SuperScript VILO Master Mix (Thermo-Fisher Scientific). TaqMan Fast Advanced Master Mix (Thermo-Fisher Scientific) was added to cDNA along with predesigned Taqman Gene Expression Assay primers for *POLQ* (Assay ID: Hs00981375; Thermo-Fisher Scientific) and GAPDH (Assay ID: Hs99999905, Thermo-Fisher Scientific) to conduct the qRT-PCR. RNA abundance was determined by the comparative Ct method using QuantStudio 3 Design and Analysis Software (Thermo-Fisher Scientific). GAPDH was used as an endogenous control to assess relative *POLQ* mRNAs in each cell line.

### ICGC patient cohort

Pathologically confirmed prostate cancer patients with localized disease were used for this study. Fresh-frozen treatment and hormone naïve tumor specimens were obtained from the University Health Network (UHN) Pathology BioBank or from the Genito-Urinary BioBank of the Centre Hospitalier Universitaire de Québec (CHUQ). Gleason Grades were assessed by expert genitourinary pathologists (TvdK, BT, AR) using scanned hematoxylin and eosin (H&E)–stained slides. Baseline serum PSA abundance ng/mL measurements were taken at the time of diagnosis. Patients were treated with either CF external RT or RP. For RT patients, biochemical recurrence (BCR) was defined as an increase of more than 2.0 ng/mL above the nadir serum PSA abundance. For RP patients, BCR was defined as two consecutive measurements of more than 0.2 ng/mL after surgery.

### Selecting radioresistance modulator candidates

Candidate radioresistance modulators were selected based on their likelihood to affect radioresistance. Selected candidate genes were: (i) driver genes impacted highly or moderately by SNVs relying on SNPeff (RRID:SCR_005191; ref. 26) estimation (ii) dysregulated driver genes at the RNA (Supplementary Table S2) and/or protein levels (Supplementary Table S3) based on applying statistical cutoffs (FDR ≤ 0.05; iii) fusion transcripts identified in all three replicates for CF- or HF-resistant cells; (iv) genes that were directly perturbed by SV that were also dysregulated at the RNA level (two levels of indication). Overall, 291 candidates were selected for further investigation.

### Association of CNA occurrence with BCR in the ICGC cohort

CNAs were called as previously described (59). Using the entire cohort, the rate of BCR was compared between patient samples with a major-class CNA event, to those without an event or with the minor-class CNA event, by fitting Cox proportional hazard models using the survival R package v3.2-10. If the Cox proportional hazard assumption did not hold, a log-rank test or a Heinze log-rank test for imbalanced sample sizes (60) was carried out. For this analysis, only genes with a major-class CNA event in at least five patients were used.

### Patient WGS SNV detection in the ICGC cohort

Sample preparation and whole-genome sequencing (WGS) were conducted as previously described (61, 62). Raw sequencing reads aligned to the human genome reference build hs37d5 using BWA (v0.7.12 - 0.7.15; RRID:SCR_010910; ref. 20). Lane-level BAM files from the same library were merged and duplicates were marked using Picard (v1.121 - 2.8.2; ref. 22). Lane-level BAM files from the same were merged as well and no duplicates were marked.

Local realignment and base quality recalibration were done using GATK (v3.4.0 - 3.7.0; RRID:SCR_001876; ref. 23), processing tumor and normal pairs together. Individual tumor and normal sample BAM files were created and SAMtools (v0.1.19 - 1.5; RRID:SCR_002105; ref. 9) was used to correct their headers. Cross-individual contamination was evaluated using GATK ContEst (v3.4.0 - 3.7.0; RRID:SCR_001876; ref. 63) for all normal and tumor samples.

Somatic SNVs were detected using SomaticSniper v1.0.5 (RRID:SCR_005108; ref. 25) using the default parameters but setting the q option (mapping quality threshold) to one. A series of scripts provided by SomaticSniper package v1.0.5 was used to filter out possible false positives. First, a pileup Indel file was generated for both normal and tumor BAM files using SAMtools v0.1.6 (RRID:SCR_002105; ref. 9), to enable standard and LOH filtering. Second, bam-readcount (v0.8.0 dea4199) was run for false positive filtering. Lastly, the high confidence filter was used with the default parameters. In addition, identified SNVs in positions that were not covered by a minimum of 10× in normal and 17× in tumor (calculated using bedtools v2.26.0 (RRID:SCR_006646; ref. 64) were removed.

Germline variants were called using GATK (v3.4.0 - 3.7.0; ref. RRID:SCR_001876) to enable the filtering of somatic variants. GATK HaplotypeCaller was first used considering the normal and tumor BAM files together, followed by GATK VariantRecalibrator and GATK ApplyRecalibration. In Germline variant, filtering was performed by removing somatic and ambiguous variants (i.e*.*, with more than one alternate base). SNVs were filtered using the Perl library Bio::DB::HTS::Tabix v2.10 using public germline databases and the germline variants detected in all patient samples.

The final SNVs were annotated by ANNOVAR v2017-07-16 (RRID:SCR_012821; ref. 65) using the RefGene database. Only non-synonymous, stop-loss, stop-gain, and splice-site SNVs were considered.

### *POLQ* RNA associations in the ICGC cohort

RNA sequencing (RNA-seq) was performed as previously described (66). Patient mRNA TPM values for each gene were used for all analysis steps. Linear regression was used to associate *POLQ* RNA abundances in 140 patients with CNA [identified previously (59)] or SNV events (called as described above) in clinically relevant genes (i.e., significant genes following the tests listed above: differences in CNA frequencies between RP and RT patient groups and/or association of CNA occurrence with BCR). FDR was used to control multiple testing separately for *POLQ*-RNA associations with SNVs and with CNAs. Spearman correlations were calculated to evaluate the relationship among RNA abundances of clinically relevant genes in the same group of 140 patients, and FDR was used to correct for multiple testing. Spearman correlations were calculated to evaluate the relationship between *POLQ* RNA abundances and the following clinical features: age, Gleason grades, T category, and serum PSA abundance. FDR was applied to control for multiple testing.

### Testing *POLQ* inhibition signature

RNA sequencing and proteomics profiling was performed as previously described for four previously published prostate cancer datasets: International Cancer Genome Consortium (ICGC; refs. 66, 67), The Cancer Genome Atlas (TCGA; ref. 68), Khoo (69), and Houlahan (70). For RNA sequencing, log_2_ transformed transcript per million (TPM) was utilized for all analyses. For proteomics, LFQ intensities were used for protein quantification as previously described (69) with missing values imputed using the Perseus method. Protein quantification was log_2_ transformed prior to further analysis. Pearson correlation with significance was calculated for each target RNA or protein abundance and *POLQ* RNA abundance. For target protein abundance to *POLQ* RNA abundance correlation, patient-matched samples were utilized, and pairwise complete observations were included. Fold change was calculated for each target using the log_2_ transformed mean RNA or protein abundance in tumor versus normal tissue samples. Statistical significance was calculated using a two-sided *t* Test. FDR was applied to control for multiple testing. Notably, *POLQ* was not detected within our proteomics analysis at either the cell line or patient level. This is not surprising given the large *POLQ* protein size and the lack of *POLQ* identification by others, according to the Cell Map database (71).

### NCCS patient cohort

The National Cancer Centre Singapore (NCCS) cohort includes 185 patients who were diagnosed with biopsy-proven localized prostate adenocarcinoma. These patients underwent treatment RT or androgen deprivation therapy (ADT) in combination with RT at the NCCS from April 21, 2011, to November 30, 2021, with available tissue for molecular profiling. RT was delivered using either intensity-modulated RT (IMRT) or IGRT. Patients with National Comprehensive Cancer Network–defined intermediate- or high-risk prostate cancers received either short-course (≤6 months) or long-course (>6 months) ADT in combination with RT. In the definitive setting, IMRT/IGRT was delivered at doses of 74 to 78 Gy in 37 to 39 fractions, 57 to 60 Gy in 19 to 20 fractions, or 36.25 to 37.5 Gy in five fractions. BCR was defined based on the Phoenix criteria where nadir PSA + 2 ng/mL after undergoing RT. Patients whose sample quality failed the microarray sample quality control score were excluded from the analyses.

The study was explained to the patients, who were then invited to participate. Written informed consent was obtained from all participants. The study was approved by the SingHealth Centralised Institutional Review Board (CIRB) and conducted in accordance with the IRB protocol no. 2019/2177.

### NCCS tumor sampling and RNA abundance profiling

Treatment-naïve tumors from the NCCS cohort were sampled from formalin-fixed, paraffin-embedded diagnostic biopsies following review by an expert genitourinary pathologist to delineate the tumor region bearing the highest Gleason grade group and cellularity was at least 70%. Two 2-mm cores were obtained, and RNA extraction, cDNA amplification, and microarray hybridization for gene expression profiling were performed. RNA abundance profiling for the NCCS cohort was performed in accordance with the protocols used in a Clinical Laboratory Improvement Amendments (CLIA)-certified clinical operations laboratory (Veracyte Inc.). RNA data were obtained using the Human Exon 1.0 ST oligonucleotide microarray (ThermoFisher), which measured the expression of 46,050 genes and non-coding RNA transcripts. Microarray data were normalized using Single Channel Array Normalization.

### *POLQ* RNA associations in validation cohorts

The RNA abundances of *POLQ* in the NCCS cohort were divided into lower, middle, and higher tertiles by using *ntile* v1.0.10 function of the dplyr R package (72). Then, the rate of BCR was compared between patient samples with a high *POLQ* RNA abundance to those with low or intermediate RNA abundance by fitting Cox proportional hazard models using the survival R package v3.2-10. Spearman correlations were calculated to evaluate the relationship between *POLQ* and *BRCA2* RNA abundance. The raw data used for the analysis can be found in Supplementary Table S4.

In TCGA (68), log_2_ transformed TPM RNA abundance was used for associations. RNA abundances were associated with grade groups and T-categories in 483 patients using ordinal logistic regression relying on the MASS package in R v7.3.60 (73). Patient-matched samples were utilized to test differences between tumor and normal RNA abundances using a paired *t* test.

Gene counts generated by Ross-Adams and colleagues (74) were downloaded from GEO, accession number GSE70770. Following counts per million normalization and log_2_ transformation using edgeR v4.0.16 (RRID:SCR_012802; refs. 75, 76) , the rate of BCR was compared between patient samples with a high *POLQ* RNA abundance (upper 20%), to those with low or intermediate RNA abundances (lower 80%) by fitting a Cox proportional hazard model.

### Visualization and statistical analysis

Visualizations and statistical analysis for this paper were carried out in the R statistical environment v4.2.0. Visualizations were generated using the BPG package v7.0.3 (77).

### Data availability

DU145 and 22Rv1 cell line genomic and transcriptomic data have been deposited to SRA under the accession number PRJNA1008743. DU145 cell line miRNA NanoString data have been deposited to GEO under the accession number GSE241078. DU145 cell line proteomic raw data have been deposited to the Mass Spectrometry Interactive Virtual Environment (MassIVE) with the following MassiVE ID: MSV000092485 and FTP link: ftp://massive.ucsd.edu/MSV000092485/. The raw NCCS patient data used in this study are available in the Supplementary Material.

## Results

### Radioresistance induces widespread genomic instability

**Figure 1:**
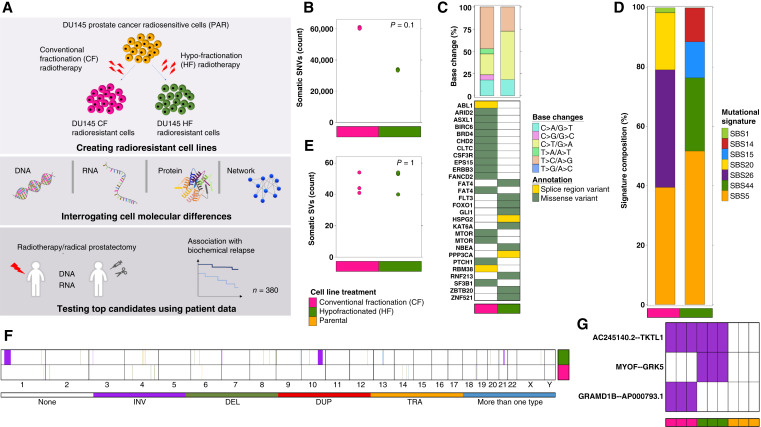
The genomic landscape of CF- and HF-resistant prostate cancer cells. **A,** Schematic of experimental design and workflow. **B,** Somatic SNV count for CF- vs. HF-resistant cells. CF-resistant cells gained twice more SNVs than HF (*P* = 0.1; Mann–Whitney *U* test). **C,** SNVs in cancer driver genes. Presented are all driver genes that are predicted to be strongly influenced by SNVs. Considered are SNVs that were identified in all three replicates for each cell type. Top, single-base substitution types. Bottom, the predicted annotation. **D,** Gained SNVs converged on partly similar cancer mutational signatures. Most signatures of known etiology, irrespective of the treatment schedule, are associated with defective DNA mismatch repair. Signature etiologies: SBS5, unknown; SBS26 and SBS15, defective DNA mismatch repair; SBS1, spontaneous deamination of 5-methylcytosine; SBS14, concurrent polymerase epsilon mutation and defective DNA mismatch repair; SBS20, concurrent *POLD1* mutations and defective DNA mismatch repair; SBS44, defective DNA mismatch repair. **E,** Somatic SV counts for CF- and HF-resistant cells. The number of somatic SVs is similar between CF- and HF-resistant cells (*P* = 1; Mann–Whitney *U* test). **F,** Distinct SVs in CF-resistant cells compared to HF across the genome. Considered are SVs that were identified in all three replicates for each cell type. Chromosome numbers are presented on the *x*-axis. The colored lines represent types of SVs: DEL, deletion; DUP, duplication; INV, inversion; TRA, translocation. **G,** Fusion transcripts that were identified in either CF- and/or HF-resistant cells. Purple, the fusion transcript was identified; white, the fusion transcript was not identified. The results are presented for three replicates for each cell line.

To characterize the molecular determinants of radioresistance in prostate cancer, we exploited isogenic DU145 radioresistant cells created by mimicking CF and HF treatment schedules (17, 18). We performed DNA WGS, RNA sequencing, and whole-cell and organellar proteomics (Fig. 1A) to characterize the molecular response to radiation. We first evaluated the number of mutations induced by radiation across the entire genome. CF-resistant cells acquired ∼60,000 somatic SNVs per biological replicate after RT—mutations absent in the parental cells (Fig. 1B). In contrast, HF-resistant cells acquired fewer, at ∼30,000 new SNVs (*P* = 0.1; Mann–Whitney *U* test; Fig. 1B).

Relative to the typical few 1,000 SNVs in newly diagnosed localized prostate cancers (61, 78), the number of RT-associated point mutations was very large. To validate this result, we next created CF-radioresistant 22Rv1 cells and again performed DNA sequencing. We saw a comparable increase in SNV mutations/Mbp of genomic DNA in this second model system (Supplementary Fig. S1A). CF-resistant cells experienced widespread thymine to cytosine mutations, whereas cytosine to thymine predominated in HF-resistant cells (Supplementary Table S5). Both CF- and HF-resistant cells contained multiple driver-affecting SNVs, including two separate point mutations in *MTOR* in CF-resistant cells (Fig. 1C).

Independent of fractionation, mutations occurring in radioresistant cells showed strong signatures of defective DNA mismatch repair, but there were fractionation-dependent differences in the signature types: SBS20 and SBS26 for CF-resistant cells and SBS14, SBS15, and SBS44 for HF (Fig. 1D). Upregulation of proliferating cell nuclear antigen (*PCNA*) transcript and dysregulation of polymerase delta (*POLD1*) isoforms were detected in CF-resistant cells (Supplementary Fig. S2), consistent with the association of the CF-exclusive signature SBS20 with *POLD1* (79–81). We again confirmed these results in CF-radioresistant 22Rv1 cells (Supplementary Fig. S1B). SBS5 was conserved between CF and HF (Fig. 1D).

Radioresistance was also associated with increased genomic instability in double-stranded breaks, with significant additional SV (Fig. 1E). While the total number of SVs was similar between CF- and HF-resistant cells, there were no common SV regions. (Fig. 1F). No SVs appeared to directly perturb known cancer driver genes, although a subset was associated with gene expression changes (Supplementary Fig. S1C). For example, a chromosome 10 inversion that disrupted *MYOF* and *GRK5* in HF-resistant cells led to the presence of *MYOF*-*GRK5* fusion transcripts solely in these cells (Fig. 1G; Supplementary Table S6).

### Extensive transcriptional and post-transcriptional responses to RT

**Figure 2:**
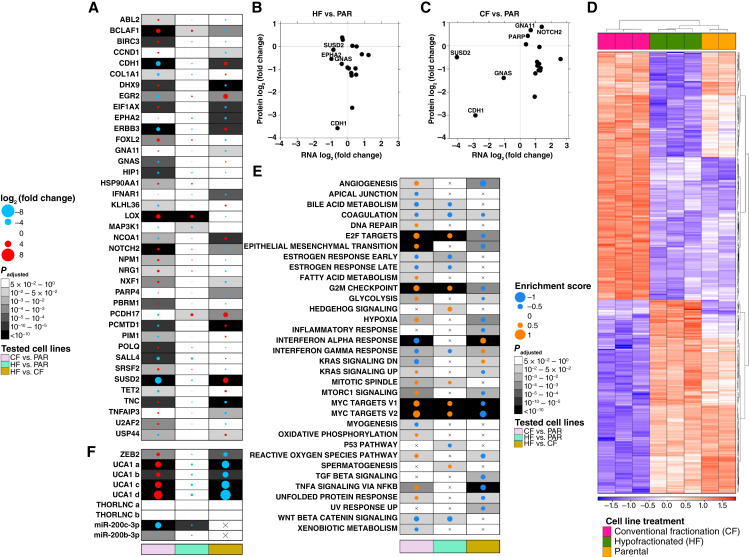
Transcriptomic signatures of cancer-related genes in radioresistant cells. **A,** The differences in RNA abundances of driver cancer genes. The most abundant transcript out of all transcripts per gene was taken for visualization. **B–C,** The differences in RNA abundances of affected driver gene, compared to their abundance differences at the protein level. **B,** Differences between CF-resistant cells and the parental cells. **C,** Differences between HF-resistant cells and the parental cells. **D,** The RNA abundance profile of cancer hallmark genes in CF-resistant cells is distinct from HF-resistant cells and the parental profiles, which are relatively similar. The heatmap presents normalized counts of RNA isoforms that were significantly differentially abundant in CF-resistant cells compared to HF. Red, high abundance; blue, low abundance. For visualization, RNA abundances as a function of log_10_ were converted to *z*-scores. **E,** Enrichment analysis of hallmark gene sets. The dot size represents the enrichment score and the dot color represents the directionality: for CF vs. PAR, HF vs. PAR, and HF vs. CF tests, orange represents upregulation toward CF-resistant cells, HF-resistant cells, and HF-resistant cells. **F,** The differences in RNA abundances of the lncRNA *UCA1*, the miRNAs 200c-3P and 200b-3P, and *ZEB2* (the target of the miRNAs 200c-3P and 200b-3P). For *UCA1* transcripts, the small letters represent different isoforms. In **A** and **F,** the dot size represents the log_2_ (fold change) size and the dot color represents the directionality: for CF vs. PAR, HF vs. PAR, and HF vs. CF tests, red represents upregulation toward CF-resistant cells, HF-resistant cells, and HF-resistant cells. For all figures , three RNA-seq replicates were used for the radioresistant cell lines and two for the parental line. In **B–C**, three replicates were used for all cell types.

We next considered global RNA abundance profiling, where similar numbers of transcripts were detected in all groups (Supplementary Fig. S3A; Supplementary Table S7) with similar global patterns between groups (Supplementary Fig. S3B). Hundreds to thousands of specific transcripts were differentially abundant between parental and radioresistant groups (Supplementary Fig. S3C–S3E). Consistent with DNA sequencing, the transcriptome of HF-resistant cells was less perturbed than that of CF-resistant cells (271 vs. 1,416 significant transcripts at the level of FDR ≤ 0.05; Supplementary Fig. S3F). This was particularly evident in cancer-driver genes, in which almost all changes occurred in CF-resistant but not HF-resistant cells (Fig. 2A; Supplementary Table S1). Most of these RNA changes translated to differential protein abundance in HF (Fig. 2B) or CF (Fig. 2C) cells. Most notable of these was *CDH1*, a classic prostate cancer driver gene that its loss has been previously associated with radioresistance (82). Intriguingly *CDH1* was downregulated at the RNA level only in CF-resistant cells but not in HF-resistant cells (Fig. 2A). By contrast, it was significantly downregulated in protein for both fractionation schedules, suggesting differing mechanisms (Fig. 2B and C). Supporting this, cancer hallmark genes were preferentially changed in radioresistant cells (Supplementary Fig. S3G) and distinguished fractionation schedules (Fig. 2D) much more clearly than did the global transcriptome (Supplementary Fig. S3B). Multiple pathways were specifically dysregulated in radioresistant cells (Fig. 2E), with CF-resistant cells being more disrupted, consistent with genomic and univariate transcriptomic data. Similar numbers of significant RNA abundance changes in drivers occurred in CF-resistant 22Rv1 cells.

Furthering this concept of fractionation-specific gene-regulatory pathways, we considered long non-coding RNAs (lncRNA). The lncRNA *UCA1* has been associated with CF radioresistance (17), and consistently here we identified four *UCA1* isoforms heavily upregulated in CF-resistant cells, but none in HF-resistant cells (Fig. 2F). The RNA counts of the oncogenic lncRNA THOR (83) were very low in all samples and did not yield significant changes following RT (Fig. 2F). The lncRNA *SCHLAP1*, which promotes aggressive prostate cancer (84), was not detected in any of the samples.

To further characterize post-transcriptional signaling responses to radiation, we then quantified global microRNAs (miRNA) abundance in each condition. Similar miRNA abundance patterns were observed between groups (Supplementary Fig. S3H and S3I). We identified 23 differentially abundant miRNAs (Supplementary Fig. S3J; Supplementary Table S8). CF- and HF-exclusive miRNAs targeted 14% and 11% of the exclusive CF and HF dysregulated genes, respectively (Supplementary Fig. S3K), suggesting that RNA abundance differences in CF versus HF are at least in part caused by differential regulation of miRNA “master regulators”. We highlighted the miRNA target *ZEB2*, which promotes radioresistance via recombination-dependent DNA repair (85) and its regulation by *miRNA-200b-3p* and *miRNA-200c-3p* (Fig. 2F). Taken together, these data demonstrate wide-spread, fraction-specific modes of radioresistance that are significantly shaped by post-transcriptional regulatory processes.

### Protein subcellular responses to RT are fractionation dependent

**Figure 3:**
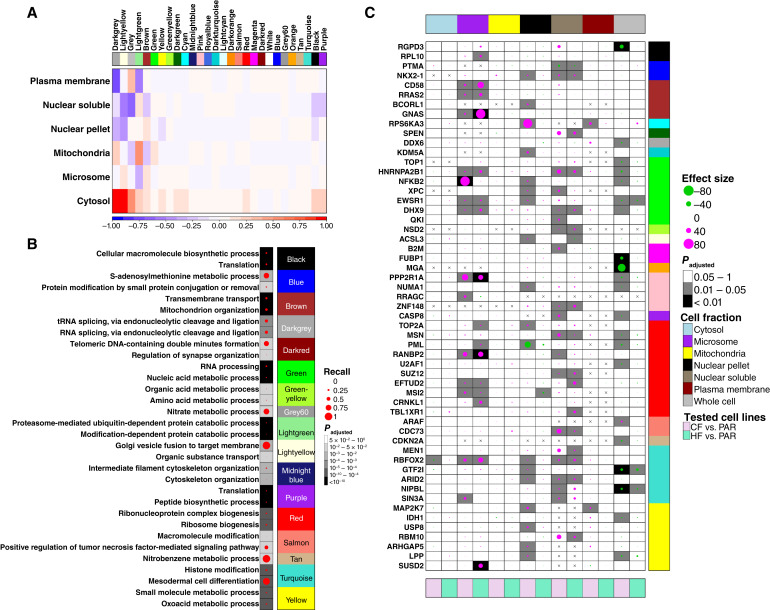
Fractionation-dependent protein profiles in radioresistant cells. **A,** The difference in relationships of consensus-module eigengenes and cellular fractions between CF- and HF-resistant cells. The colors represent the difference in correlations between consensus module eigengenes and a specific subcellular fraction of HF-resistant cells compared to CF: Blue, a higher correlation in CF-resistant cells; Red, a higher correlation in HF-resistant cells. At the top, each color represents a module, which is a detected group of positively correlated genes that are highly interconnected. **B,** Gene ontology enrichment of module genes for biological processes. The top two enrichments for each module are presented. **C,** Differences in protein abundances of driver, across different subcellular fractions and in whole cell lysates. Main, protein Cohen’s d effect sizes of significant proteins across cell fractions. Only significant changes at the level of FDR ≤ 0.025 were plotted. The dot color is the directionality: magenta and green represent upregulation and downregulation, respectively, toward CF- or HF-resistant cells. Right, the consensus modules that each gene was assigned to. For all figures, three replicates were used for CF- and HF- resistant cells. For the parental cells, at least two replicates were used.

To better evaluate post-transcriptional regulation in radioresistance, we performed subcellular fractionation to enrich for distinct organelles (47), which were independently analyzed by proteomics (Supplementary Tables S9 and S10). Using network analysis, we identified 28 gene modules (groups of co-expressed genes among two data sets) with disparate abundance patterns across fractions (Fig. 3A). Most were more abundant in the cytosols of HF-resistant cells, but conversely were more abundant in other subcellular compartments of CF-resistant cells. Several of these fraction-biased networks were preferentially associated with specific pathways (Fig. 3B). For example, genes involved in RNA splicing (“dark gray” module) were preferentially cytosolic in HF-resistant cells but nuclear in CF-resistant cells.

To understand how specific proteins were associated with radioresistance, we performed differential proteomic abundance analysis in each fraction and in whole cell lysates. Consistent with our DNA and RNA findings, CF-resistant cells were significantly more perturbed in whole cell lysates (Supplementary Fig. S4A and S4B). This difference was driven by all subcellular fractions (besides the mitochondria, where no dysregulation was observed for both cell types) and especially by the nucleus and the plasma membrane (Supplementary Fig. S4C–S4N). Across fractions, a total of 132 cancer driver genes showed proteomic dysregulation (Fig. 3C; Supplementary Table S3), larger than at either the DNA or RNA levels (Supplementary Fig. S5C). As a specific example, CD44 has been proposed as a potential driver of radioresistance (18). Its transcriptional dysregulation was restricted to five isoforms identified in CF-resistant cells and four in HF-resistant cells (Supplementary Fig. S5A); these isoforms have distinctive functional roles (86). Similarly, its protein dysregulation was exclusively reflected in the nuclear soluble fraction (Supplementary Fig. S5B). This highlights the isoform- and subcellular compartment-specific changes induced by radioresistance.

Consistent with the presence of significant post-transcriptional and translational components of radioresistance, RNA and protein abundances were only weakly correlated [Supplementary Fig. S5C (left)]. The median correlation was 0.15, lower than the typical 0.3 observed in primary prostate cancers (67). By contrast, these correlations strengthened significantly when only cancer hallmark genes were considered, most prominently in CF cells [Supplementary Fig. S5C (right)]. This suggests that transcriptional regulation was an important mode of radioresistance for cancer-related genes. Indeed, a large group of driver genes that were transcriptomically dysregulated were proteomically dysregulated in one or more specific subcellular compartments (Supplementary Fig. S5D). Fully 28% of drivers that showed RNA and protein changes in CF cells were members of the green module (Supplementary Fig. S5D), suggesting that specific network modules may reflect specific regulatory patterns. Taken together, these data demonstrate that radioresistance significantly reshapes post-transcriptional, translational, and post-translational processes.

### Primary patient data highlights *POLQ* as a mediator of radioresistance

**Figure 4:**
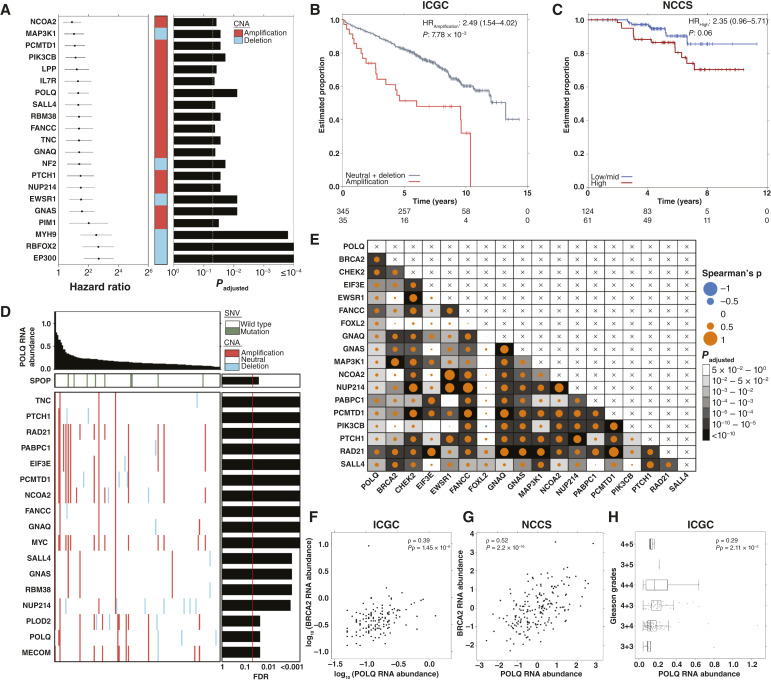
Characteristics of radioresistance modulator candidates in primary prostate tumors. **A,** Significant associations (FDR ≤ 0.05) between CNAs in candidate genes and biochemical recurrence, as shown by fitting Cox proportional hazard models in ICGC. **B,***POLQ* amplification is associated with biochemical relapse in ICGC (Cox proportional hazard model; FDR = 7.78 × 10^−3^). **C,** High POLQ RNA abundance is associated with BCR following an RT treatment in NCCS (Cox proportional hazard model; *P* = 0.06). **D,** Increased RNA abundance of *POLQ* is associated with a high mutation ratio in clinically relevant candidate genes based on linear regression in ICGC. *P*_adjusted_ levels after FDR correction are presented on the (right). **E,** Significant correlations between RNA abundances of clinically relevant candidates and *POLQ* RNA abundance. **F** and **G,** Strong correlation between *POLQ* and *BRCA2* RNA abundance in ICGC (**F**) and NCCS (**G**). **H,** Increased RNA abundance of *POLQ* is associated with high Gleason grades. In **F–H,** ρ: Spearman correlation. *P*_ρ_: the *P*_adjusted_ after FDR.

To determine which genes associated with radioresistance in pre-clinical model systems might influence primary patient phenotypes, we interrogated the 382 patient ICGC PRAD-CA dataset. This cohort includes patients treated with curative intent, either by surgery or RT (Table 1). Of 291 preclinical candidates selected based on the cell line investigations (“Materials and Methods”; Supplementary Table S11), 28 were prognostic of BCR; (Fig. 4A). Out of these 28 genes, we chose to focus on *POLQ*, due to its well-known role in double-strand DNA (dsDNA) break repair (87). In cell lines, *POLQ* transcripts were upregulated strongly in CF resistant cells (Fig. 2A) and modestly in HF (Supplementary Table S2). Amplification of *POLQ* was associated with significantly worse prognosis in treatment-naïve prostate cancer [Fig. 4B; hazard ratio (HR) = 2.49; FDR = 7.78 × 10^−3^; confidence interval (CI) = 1.54–4.02]. We validated this finding by demonstrating an increased association of POLQ RNA abundance with BCR in two independent cohorts: (i) the NCCS cohort of 185 treatment-naïve prostate cancers treated with RT (Fig. 4C; Table 1; HR = 2.4; *P* = 0.06; CI, 0.96–5.71; ii) a cohort by Ross-Adams et al. with 93 patients treated with RP (Supplementary Fig. S6A; HR = 1.9; *P* = 0.06; CI, 0.93–3.70).

**Table 1:** Prostate cancer cohort characteristics

	ICGC	NCCS
	Median (range)	
Age at treatment	66 (42–83)	71 (66–75)
Pretreatment serum PSA abundance (ng/mL)	7.2 (0.7–39.5)	16.2 (8.6–40.7)
Gleason score	*N*	
6	65	6
7	305	109
8	8	34
9	2	36
NA	2	—
cT category	*N*	
T1	180	75
T2	202	48
T3	—	60
T4	—	2
Biochemical relapse	*N*	
Yes	127	21
No	253	165
NA	2	—
RNA data	*N*	
Available	140	185
Not available	242	—
Treatment	*N*	
RT only	151	43
RT + short-term ADT	—	42
RT + long-term ADT	—	100
RP	231	—

Abbreviations: ADT, androgen deprivation therapy; RP, radical prostatectomy; RT, radiotherapy.

To understand the molecular consequences of *POLQ* dysregulation, we evaluated the (74) association of *POLQ* RNA abundance with known prostate cancer drivers and prognostic biomarkers in ICGC PRAD-CA (61, 62, 66, 67). High *POLQ* RNA abundance was associated with increased mutation rates in many preclinical candidates and known drivers, including *MYC* and *RAD21* (linear regression, FDR ≤ 0.05; Fig. 4D; Supplementary Fig. S7). Increased *POLQ* RNA abundances were also associated with amplifications in *POLQ* itself, suggesting that the dysregulation in *POLQ* at the RNA level is driven, at least partly, by *POLQ* amplification. Positive RNA–RNA correlations were widespread between *POLQ* and multiple cancer driver genes (Fig. 4E) most notably *BRCA2* (Fig. 4F). We validated this finding in the same 185 patient-independent NCCS cohort (Fig. 4G). *POLQ* RNA abundances were also significantly higher in tumor versus normal samples (Supplementary Fig. S6B) linked to pathologic T-categories (Supplementary Fig. S6C) and closely associated with grade in two independent cohorts (Fig. 4H; Supplementary Fig. S6D; Supplementary Table S12). Collectively, we revealed a widespread association of *POLQ* with somatic and clinical features of prostate tumors that marked *POLQ* as the top radioresistance modulator candidate.

**Figure 5:**
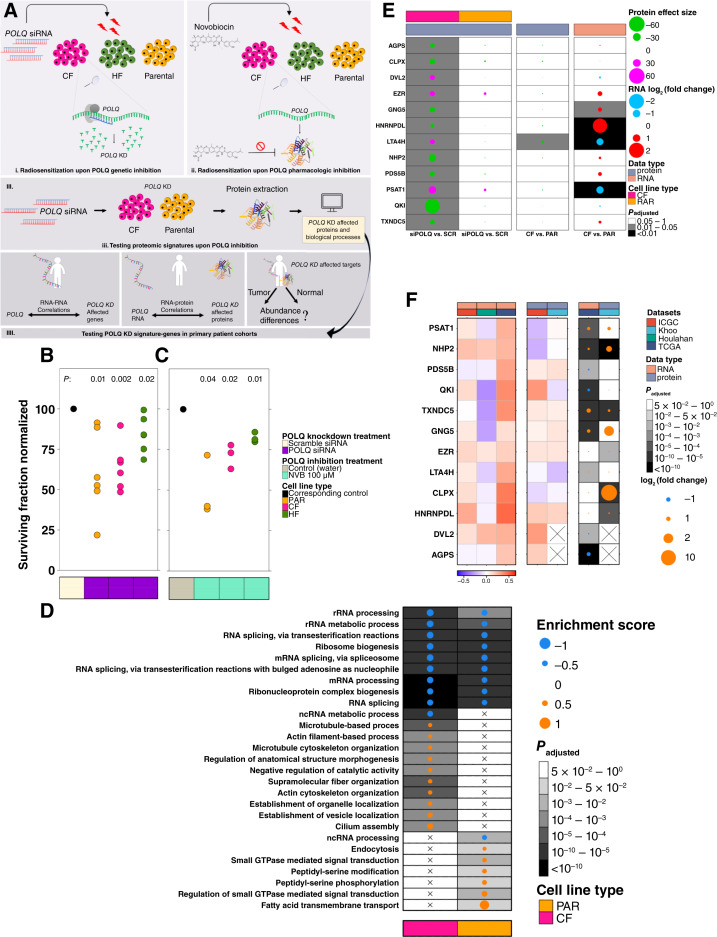
The proteome signature upon *POLQ* inhibition. **A,** Experimental design schematic. **B,** Genetic *POLQ* inhibition. The parental, CF-resistant, and HF-resistant cells were treated with either *POLQ* siRNA or scramble siRNA as the control. Significant radiosensitization was observed in all cell lines upon *POLQ* knockdown (*P* = 0.01, 0.002, and 0.02 for the parental, CF-resistant, and HF-resistant cells, respectively; paired *t* test). Three biological replicas of 4,000 cells per well and three of 6,000 cells per well were considered for each sample. **C,** Pharmacologic POLQ inhibition. CF-resistant, HF-resistant, and the parental cells were treated with 100 μmol/L novobiocin to achieve POLQ inhibition (Nvb 100 µmol/L). Significant radiosensitization was observed in all DU145 cell lines upon POLQ inhibition (*P* = 0.04, 0.02 and 0.01, for the parental, CF-resistant, and HF-resistant cells; paired *t* test). In **B** and **C,** both the control and the treated cells were irradiated with 0 or 4 Gy in two fractions. The surviving fraction of the treated cells (*POLQ* siRNA or Nvb 100 µmol/L) was normalized to the surviving fraction of the corresponding control of each cell line (black dots). **D,** The (top) 10 activated and suppressed biological processes at the protein level following *POLQ* depletion, in CF-resistant cells and the parental. The dot size represents the enrichment score, and the dot color is the directionality: orange shows upregulation toward *POLQ*-treated cells. **E,***POLQ* genetic inhibition creates a proteomic signature, involving 12 affected genes in CF-resistant cells. Three replicates from each cell type were used for the analysis. Left: signature genes whose protein abundances were changed between *POLQ*-depleted cells and the control, in CF-resistant cells and the parental cells. The dot size represents the Cohen’s d effect size, and the dot color is the directionality: magenta, upregulation, and green, downregulation toward *POLQ*-treated cells. Middle: changes in protein abundances of signature genes, between CF-resistant cells and the parental. The dot size represents the Cohen’s d effect size, and the dot color is the directionality: magenta, upregulation, and green, downregulation toward CF-resistant cells. Right: changes in RNA abundances of signature genes between CF-resistant cells and the parental. The dot size represents the log_2_ (fold change) values. The dot color represents the directionality: red shows upregulation toward CF-resistant cells. **F,** Investigating signature genes (that were affected by *POLQ* inhibition in cell lines) in primary patient data. Left: RNA-RNA Pearson correlations between *POLQ* and signature genes. Middle, Pearson correlation between the abundances of *POLQ* RNA and proteins of signature genes. Right: Abundance of signature genes in normal vs. tumor samples at the RNA and protein levels. For all panels, datasets refer to the cohort names used for analysis and the data type for the type of molecule tested for signature genes (i.e., RNA or protein).

Next, to functionally validate the role of *POLQ* in radioresistance, we knocked it down in parental, CF- and HF-resistant cells using siRNA, followed by irradiation (Fig. 5A; Supplementary Fig. S8A–S8C). *POLQ* knockdown significantly radiosensitized all three cell lines (Fig. 5B). *POLQ* pharmacologic inhibition with the inhibitor novobiocin (88) yielded similar radiosensitization (Fig. 5C). Proteomic profiling of cells following siRNA-mediated *POLQ* knockdown revealed widespread changes (Supplementary Fig. S8D) and dysregulation of multiple signaling pathways (Fig. 5D; Supplementary Table S13). A signature of 12 individual strongly dysregulated proteins (FDR ≤ 0.05) defined *POLQ*-inactivation (Fig. 5E; Supplementary Fig. S8E–S8G). Opposite dysregulation of these genes in untreated CF-resistant cells suggests their involvement in radioresistance, while *POLQ* depletion reverses their dysregulation, leading to radiosensitization [Fig. 5E (right) two panels].

To validate the 12-gene *POLQ* inhibition signature (Fig. 5D), we used transcriptomic (66, 68, 70) and proteomic (67, 69) profiling of primary prostate tumors. We first calculated the RNA abundance correlations between our signature genes and *POLQ*. Significant positive correlations between all signature genes and *POLQ* were identified in TCGA, with a mix of positive and negative correlations in the other small datasets used (Fig. 5F; Supplementary Table S14). All 12 signature genes showed transcriptomic proteomic differential abundance [Fig. 5F (right)], while two were associated individually with BCR: *PSAT1* and *CLPX* deletion (Supplementary Fig. S8H and S8I). Taken together, these data establish that *POLQ* is associated with radioresistance in preclinical model systems, that its pharmacologic or genetic inhibition reverses this phenotype and that it is associated with a gene expression signature that predicts aggressive cancer

## Discussion

Radioresistance can arise following both CF and HF and is associated with aggressive disease that might lead to further adverse oncologic outcomes (15, 16). There is an urgent need to identify mechanisms and modulators of radioresistance. We characterized the proteogenomic response to radiation in prostate cancer. Remarkably, CF resulted in a far more aggressive biomolecular phenotype than did HF, with more SNVs and strong dysregulation of hallmark-cancer and driver genes at the RNA and protein levels. These extensive biomolecular reactions to CF RT can explain the significantly more aggressive phenotype observed following CF RT (18) and are consistent with clinical reports suggesting improved disease control with HF (9, 89, 90).

Ionizing RT causes hard-to-repair dsDNA breaks, resulting in mitotic catastrophe and cell death. Irrespective of fractionation, we observed immense genomic instability dictated by defective DNA mismatch repair, which can grant oncogenic advantage. These findings imply a delicate equilibrium caused by radiation between lethal dsDNA breaks and tumor-promoting mismatch mutations. Understanding this equilibrium may be the key to inventing new therapies to improve radiation sensitivity. This equilibrium may be less favorable in CF RT compared to HF. In CF RT, small radiation doses decrease chances for cancer cell death and multiple treatments enhance opportunities to acquire mutations that confer an evolutionary advantage. In contrast, HF potentially causes more severe damage to cancer cells over far fewer treatments, greatly reducing the likelihood of cells surviving and gaining mutations. Thus, our results are logical and support the use of HF RT for treating prostate cancer.

Integrating primary prostate cancer patient data, we pinpoint *POLQ* as a top candidate modulator of radioresistance beyond our model system. *POLQ* is a DNA repair enzyme that plays an essential role in microhomology-mediated end joining (MMEJ) for dsDNA break repair. The lack of important proofreading activity in *POLQ* (87, 91) renders MMEJ susceptible to mutations. *POLQ* inhibition in combination with fractionated radiation has been shown to safely promote radiosensitization in tumor cells, while avoiding damage to normal cells, both *in vitro* and *in vivo* (92–95). The safety of *POLQ* inhibition is now being tested in a human clinical trial (NCT04991480). Our results show for the first-time radiosensitization in radioresistant cells following *POLQ* inhibition. Thus, we display the potential of targeting *POLQ*, not only to achieve radiosensitization in radiation-naïve tumor cells but also to prevent or treat radio-recurrent disease. Detailed mechanistic studies to elucidate *POLQ*’s contribution to radiation response, the use of altered fractionation schedules, and *in vivo* validation studies will be integral to move toward clinical translation.

We acknowledge the limitations to our study. We primarily utilized two common prostate cancer cell lines to derive radioresistant lines. It will be ideal in the future to establish additional CF- and HF-resistant lines to better capture the molecular heterogeneity that may contribute to radioresistance. Furthermore, we plan to utilize primary prostate cancer cell lines that should more faithfully reflect the molecular and cellular phenotype of localized prostate cancer.

This study demonstrates the complex proteogenomic response of cancer cells to radiation. This response was partly fractionation dependent, demonstrating the need to suit future translational studies to the changes in fractionation. Due to the recent adoption of HF into clinics, all patients in our cohort were treated with CF RT. It would be intriguing to extend our investigation to patients treated with HF. Larger cohort sizes of RT patients will enable higher powered analysis to identify additional therapeutic targets. Ongoing proteogenomic integration will help understand the relationships among radioresistance-associated changes at the DNA, RNA, and protein levels.

## Supplementary Material

Supplementary Figure 1 Supplementary genomic data

Supplementary Figure 2 The difference in RNA abundance of a PCNA isoform (ENST00000379143.10) and POLD1 isoforms (ENST00000440232.7 and ENST00000596648.1)

Supplementary Figure 3 Supporting data for differential RNA abundance analysis

Supplementary Figure 4 Volcano plots following differential protein-abundance analysis

Supplementary Figure 5 RNA-protein relationships

Supplementary Figure 6 Validation of the association of POLQ with prostate cancer aggressiveness

Supplementary Figure 7 Association between CNA events in seven candidate genes and BCR, using a log-rank test

Supplementary Figure 8 Supporting data for POLQ inhibition effects

Supplementary Table 1 Differentially abundant transcripts

Supplementary Table 2 Differential RNA abundance of driver cancer genes, using DEseq2 with the default parameter lfcThreshold = 0

Supplementary Table 3 Differential protein abundance of driver cancer genes, following a T-test

Supplementary Table 4 NCCS raw data

Supplementary Table 5 Ratios of single-base substitution types, for the two radioresistant cell line, in 3 replicates each

Supplementary Table 6 Fusion transcript events

Supplementary Table 7 Normalized transcript counts

Supplementary Table 8 Differentially abundant miRNAs

Supplementary Table 9 Numbers of profiled proteins

Supplementary Table 10 Protein intensities

Supplementary Table 11 Top target genes used for clinical associations

Supplementary Table 12 Spearman's correlations between POLQ abundance and clinical features in patients

Supplementary Table 13 Enriched biological processes upon POLQ knockdown

Supplementary Table 14 Supporting data for Figure 5F
