# Supplementary material for: The Proteogenomics of Prostate Cancer Radioresistance
Source: Cancer Res Commun. 2024 Sep 19;4(9):2463–79. doi: 10.1158/2767-9764.CRC-24-0292 (PMC11411600; doi:10.1158/2767-9764.CRC-24-0292)
Supplement: Supplementary Figure 1 — Supplementary genomic data [file crc-24-0292_supplementary_figure_1_suppsf1.pdf]

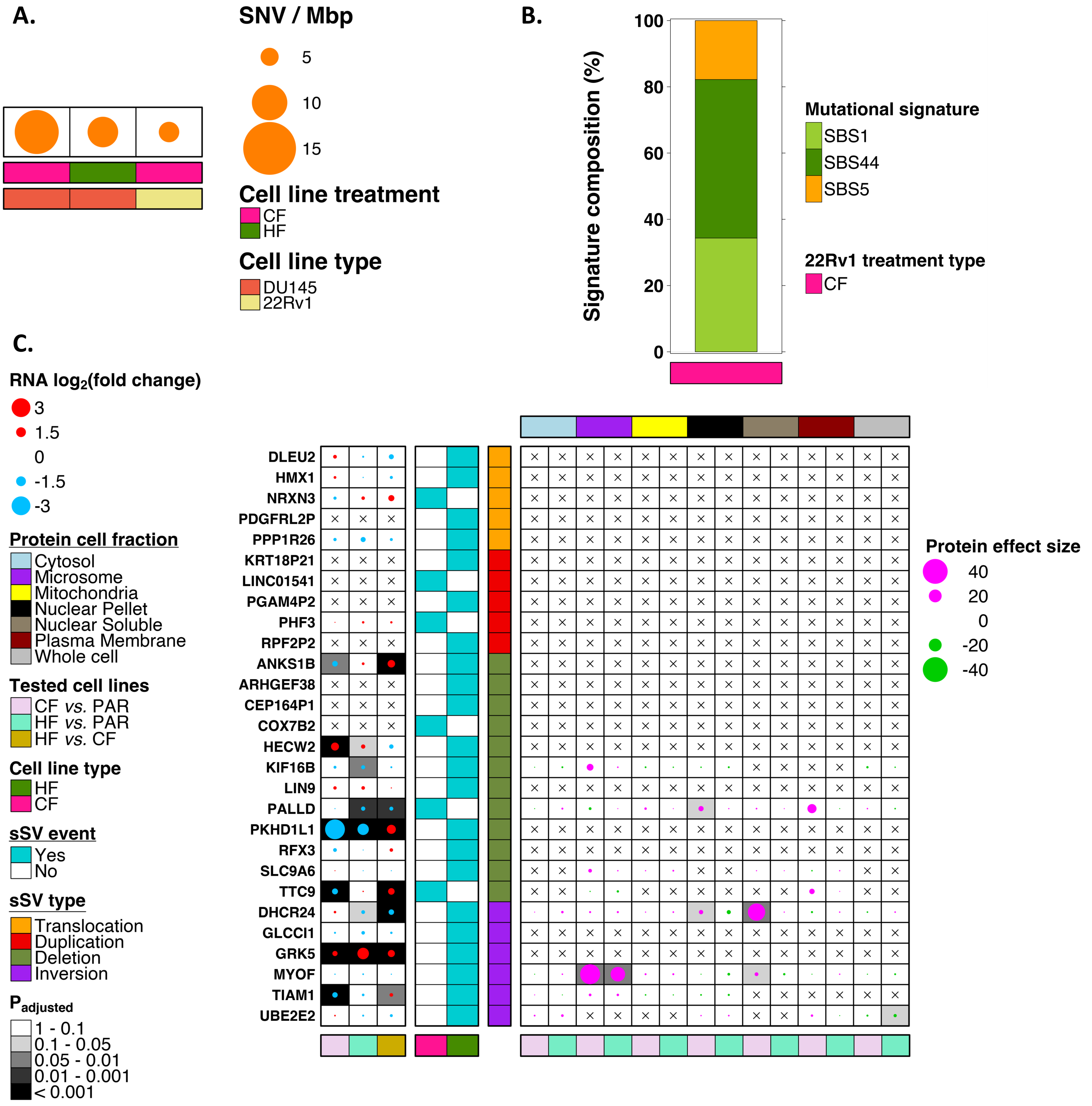

Supplementary Figure 1. A. The density of SNVs induced by radiation. For each sample, the number of SNVs that were not present in the corresponding parental cells, and were common to all replicates was divided by the number of genome sites covered by at least 20 reads in all replicates. **B.** Mutational signatures in 22Rv1 based on SNVs induced by radiation. Signature etiologies: SBS1, Spontaneous deamination of 5-methylcytosine; SBS5, unknown; SBS44 Defective DNA mismatch repair. **C.** Gene annotations of SVs and the effect on the RNA and protein abundances. Left panel, differences in transcript abundance of SVs distrusted genes,

using DEseq2 with the default parameter lfcThreshold = 0. The dot size represents the $\log_2$(fold change) size, and the dot color represents the directionality: for CF *vs.* PAR, HF *vs.* PAR, and HF *vs.* CF tests, red represents upregulation toward CF-, HF-, and HF-resistant cells respectively. For each gene, the most abundant transcript was taken. Right panel, differences in the protein levels of sSVs disrupted genes across different cell fractions. The dot size represents the Cohen's d effect size, and the dot color represents the directionality: magenta and green represent upregulation and downregulation, respectively, toward CF- or HF-resistant cells. In both the left and right panels, X represents 'non-detected'.
