# Supplementary material for: The Proteogenomics of Prostate Cancer Radioresistance
Source: Cancer Res Commun. 2024 Sep 19;4(9):2463–79. doi: 10.1158/2767-9764.CRC-24-0292 (PMC11411600; doi:10.1158/2767-9764.CRC-24-0292)
Supplement: Supplementary Figure 2 — The difference in RNA abundance of a PCNA isoform (ENST00000379143.10) and POLD1 isoforms (ENST00000440232.7 and ENST00000596648.1) [file crc-24-0292_supplementary_figure_2_suppsf2.pdf]

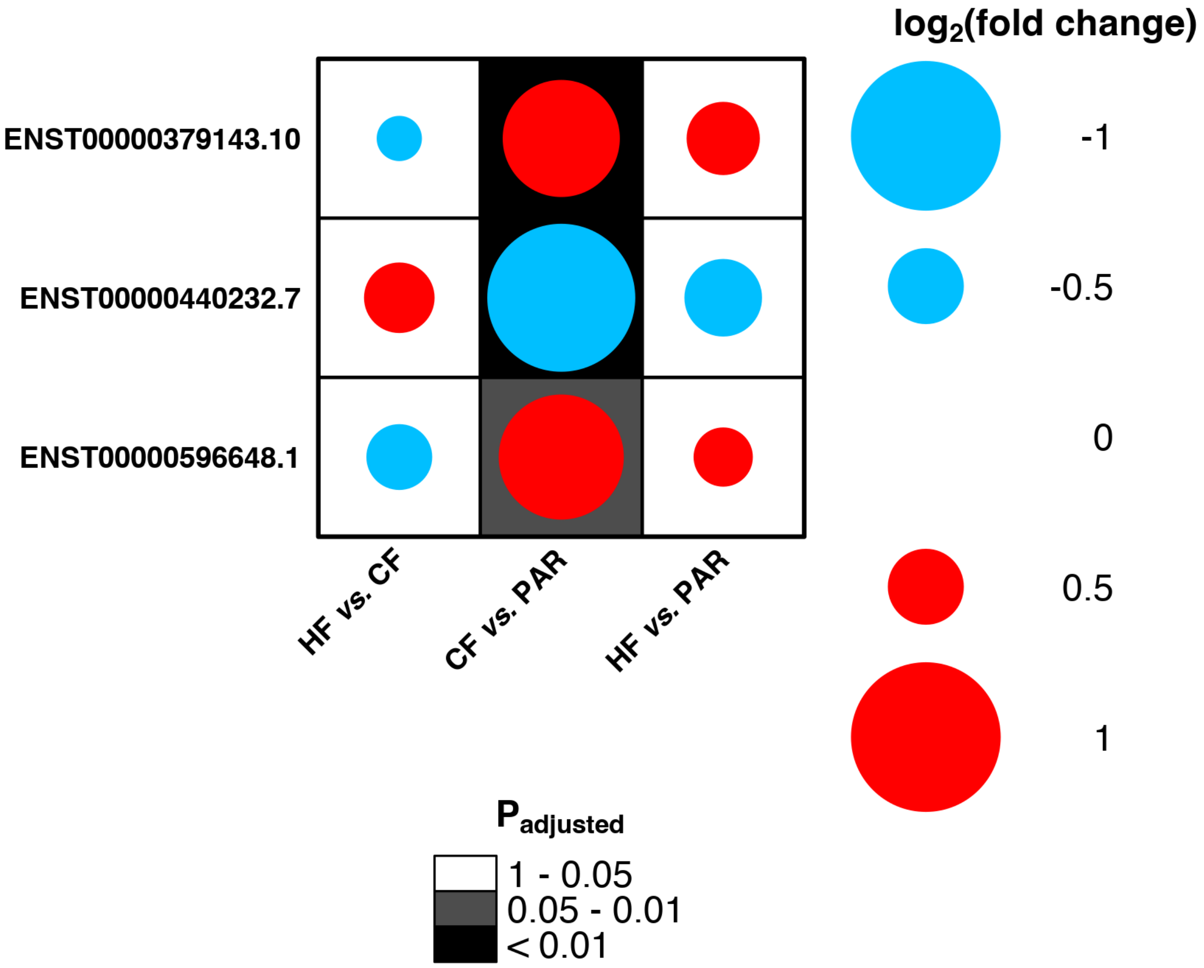

Supplementary Figure 2. The difference in RNA abundance of a PCNA isoform (ENST00000379143.10) and POLD1 isoforms (ENST00000440232.7 and ENST00000596648.1). The dot size represents the $\log_2$(fold change) size, and the dot color represents the directionality: for CF *vs.* PAR, HF *vs.* PAR, and HF *vs.* CF tests, red represents upregulation toward CF-, HF-, and HF-resistant cells respectively.
