# Supplementary material for: The Proteogenomics of Prostate Cancer Radioresistance
Source: Cancer Res Commun. 2024 Sep 19;4(9):2463–79. doi: 10.1158/2767-9764.CRC-24-0292 (PMC11411600; doi:10.1158/2767-9764.CRC-24-0292)
Supplement: Supplementary Figure 3 — Supporting data for differential RNA abundance analysis [file crc-24-0292_supplementary_figure_3_suppsf3.pdf]

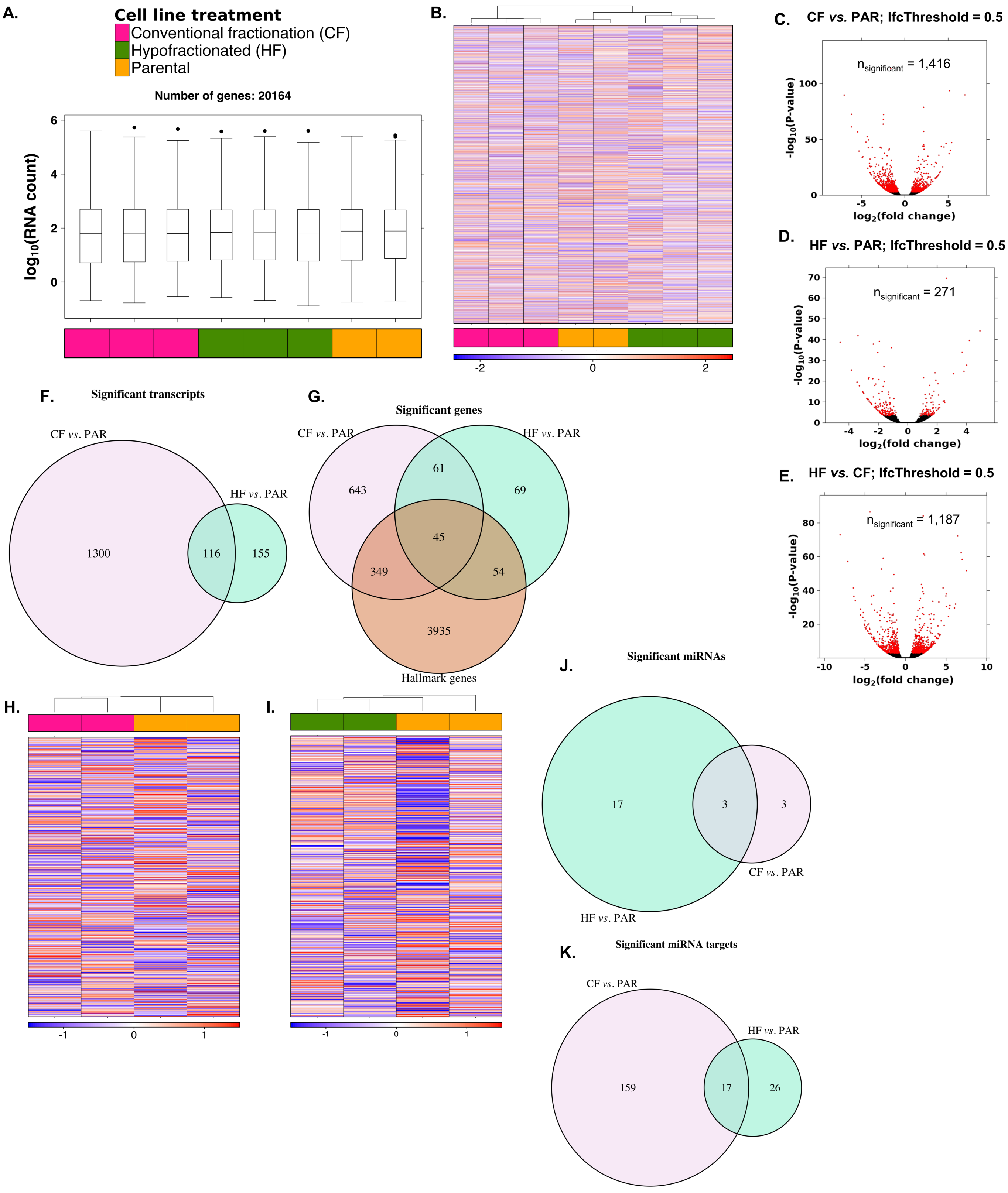

Supplementary Figure 3. Supporting data for differential RNA abundance analysis. A. The normalized transcript count distribution for each replicate. The median is shown as a solid line. **B.**

The normalized transcript counts for all replicates. Red, high counts; blue, low counts. For visualization, transcript counts as a function of $\log_{10}$ were converted to z-scores. In **A-B**, a value of 1 was added to all values in the count matrix before operating log. **C-E.** Volcano plots following differential RNA-abundance analysis, using DEseq2 with lfcThreshold = 0.5. In all panels, $n_{significant}$ refers to the number of significant transcripts (FDR $\leq$ 0.05). **F.** The overlap between significant differentially abundant transcripts in CF- and HF-resistant cells compared to the parental, following a strict test. **G.** Overlaps between CF-differentially abundant genes, HF-differentially abundant genes and the GSEA hallmark cancer gene set. For this plot, transcript names from **F** were converted into gene names, and each gene was counted only once (even if there were more than one significant transcript per gene) in CF- and HF-resistant cells. **H-I**. The normalized miRNA counts. Red, high counts; blue, low counts. For visualization, transcript counts as a function of $\log_2$ were converted to z-scores. A value of 1 was added to all values in the count matrix before operating log. **J.** The overlap between significant differentially abundant miRNAs in CF- and HF-resistant cells compared to the parental. **K.** The overlap between significant differentially abundant genes (listed in **G**) in CF- or HF-resistant cells compared to the parental, that were identified as targets for significant miRNAs (listed in **J**) for CF or HF, correspondingly.
