# Supplementary material for: The Proteogenomics of Prostate Cancer Radioresistance
Source: Cancer Res Commun. 2024 Sep 19;4(9):2463–79. doi: 10.1158/2767-9764.CRC-24-0292 (PMC11411600; doi:10.1158/2767-9764.CRC-24-0292)
Supplement: Supplementary Figure 4 — Volcano plots following differential protein-abundance analysis [file crc-24-0292_supplementary_figure_4_suppsf4.pdf]

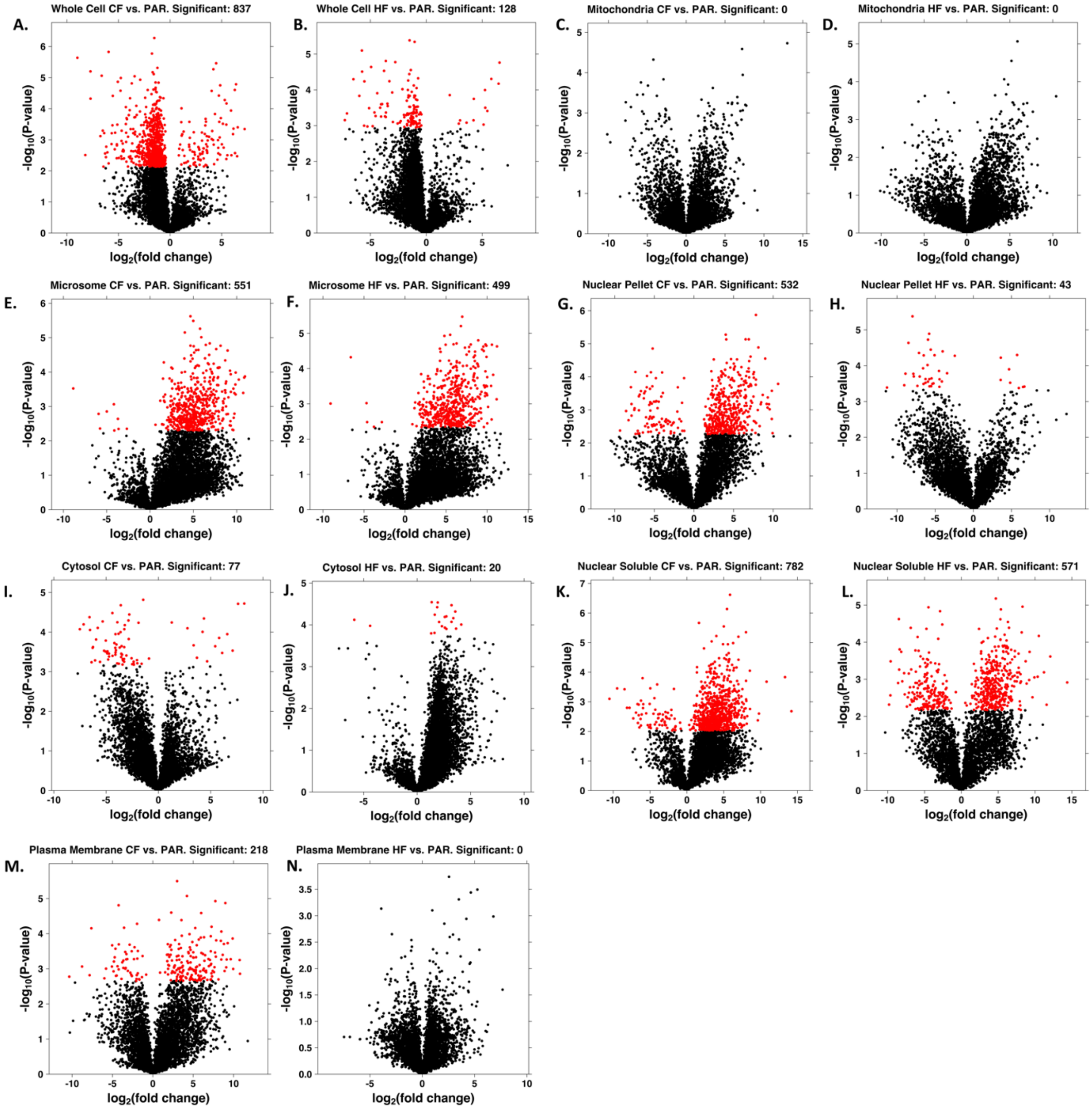

Supplementary Figure 4. Volcano plots following differential protein-abundance analysis. In all panels, significant proteins are plotted in red. Significance is defined as targets with $P_{adjusted} \leq 0.05$ following an FDR correction.
