# Supplementary material for: The Proteogenomics of Prostate Cancer Radioresistance
Source: Cancer Res Commun. 2024 Sep 19;4(9):2463–79. doi: 10.1158/2767-9764.CRC-24-0292 (PMC11411600; doi:10.1158/2767-9764.CRC-24-0292)
Supplement: Supplementary Figure 5 — RNA-protein relationships [file crc-24-0292_supplementary_figure_5_suppsf5.pdf]

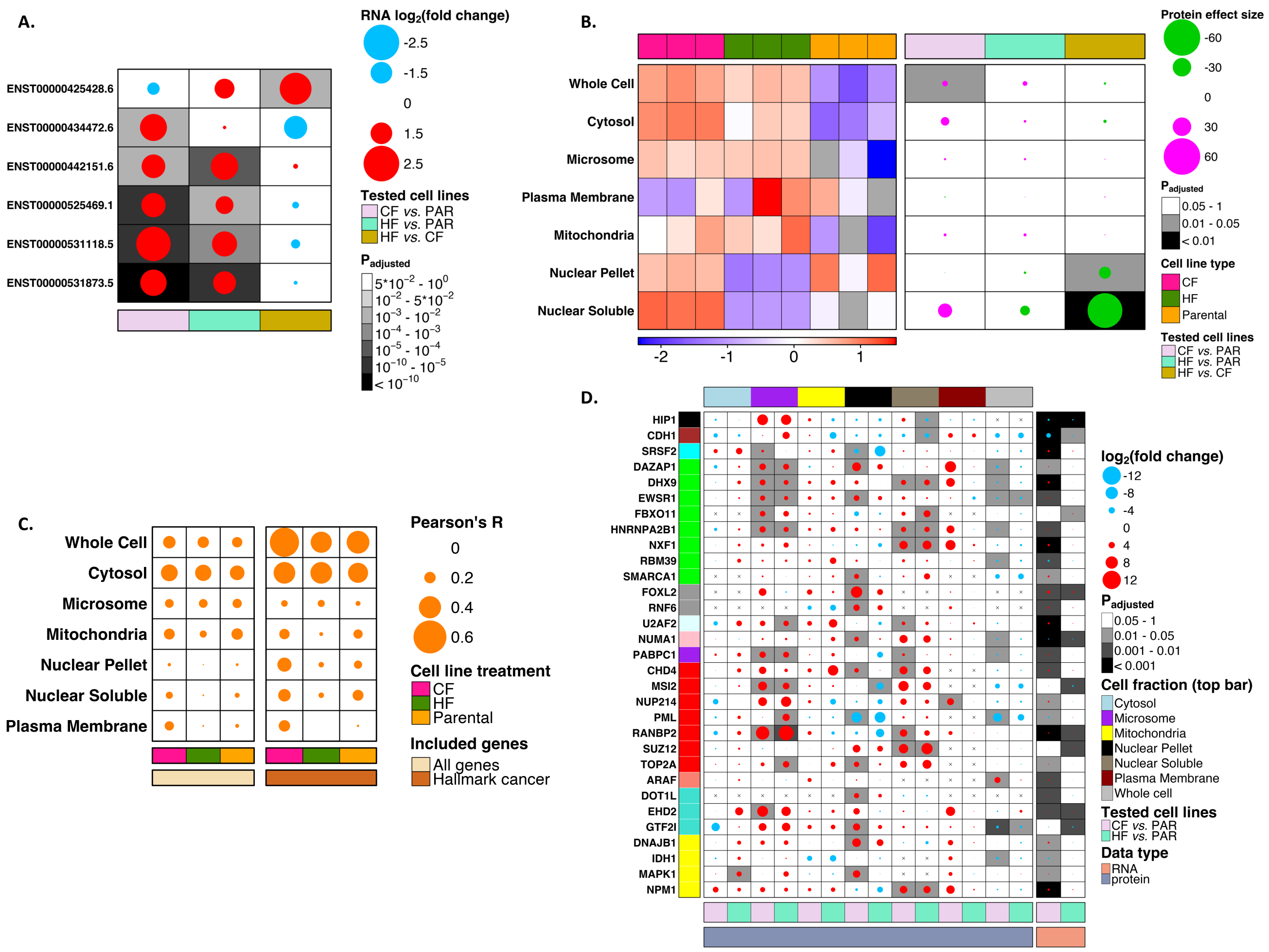

Supplementary Figure 5. RNA-protein relationships. A. Differences in RNA abundance of *CD44* isoforms. The dot size represents the $\log_2$(fold change) size, and the dot color represents the directionality: for CF *vs.* PAR, HF *vs.* PAR, and HF *vs.* CF tests, red represents upregulation toward CF-, HF-, and HF-resistant cells respectively. **B.** Differences in protein abundance of CD44 protein. Right panel: protein abundances across different subcellular fractions and cell types. Red, high abundance; blue, low abundance. For visualization, imputed protein intensities in a $\log_2$-space were converted to z-scores. Right panel: differences in protein abundance of CD44 proteins across different subcellular fractions. The dot size represents the Cohen's d effect size, and the dot color represents the directionality: for CF *vs.* PAR, HF *vs.* PAR, and HF *vs.* CF tests, magenta represents upregulation toward CF-, HF-, and HF-resistant cells respectively. **C.** Pearson correlations between the median abundance of RNA and proteins for all detected genes (left panel) or only hallmark cancer genes (right panel). Targets for which the median value was zero, were removed from correlation calculations. Protein intensities are stratified by subcellular localization. **D.** Differences in protein levels of driver genes that were affected at the RNA level in the same direction, across different subcellular fractions and in whole cell lysates. Left panel, the consensus modules that each gene was assigned to. Middle panel, protein abundance changes across cell fractions. Right panel, differences in the abundance of corresponding genes at the RNA levels, between CF- or HF-resistant cells and the parental cells. The dot size represents the $\log_2$(fold change) size, and the dot color represents the directionality: red represents upregulation toward CF- and HF-resistant cells.
