# Supplementary material for: The Proteogenomics of Prostate Cancer Radioresistance
Source: Cancer Res Commun. 2024 Sep 19;4(9):2463–79. doi: 10.1158/2767-9764.CRC-24-0292 (PMC11411600; doi:10.1158/2767-9764.CRC-24-0292)
Supplement: Supplementary Figure 6 — Validation of the association of POLQ with prostate cancer aggressiveness [file crc-24-0292_supplementary_figure_6_suppsf6.pdf]

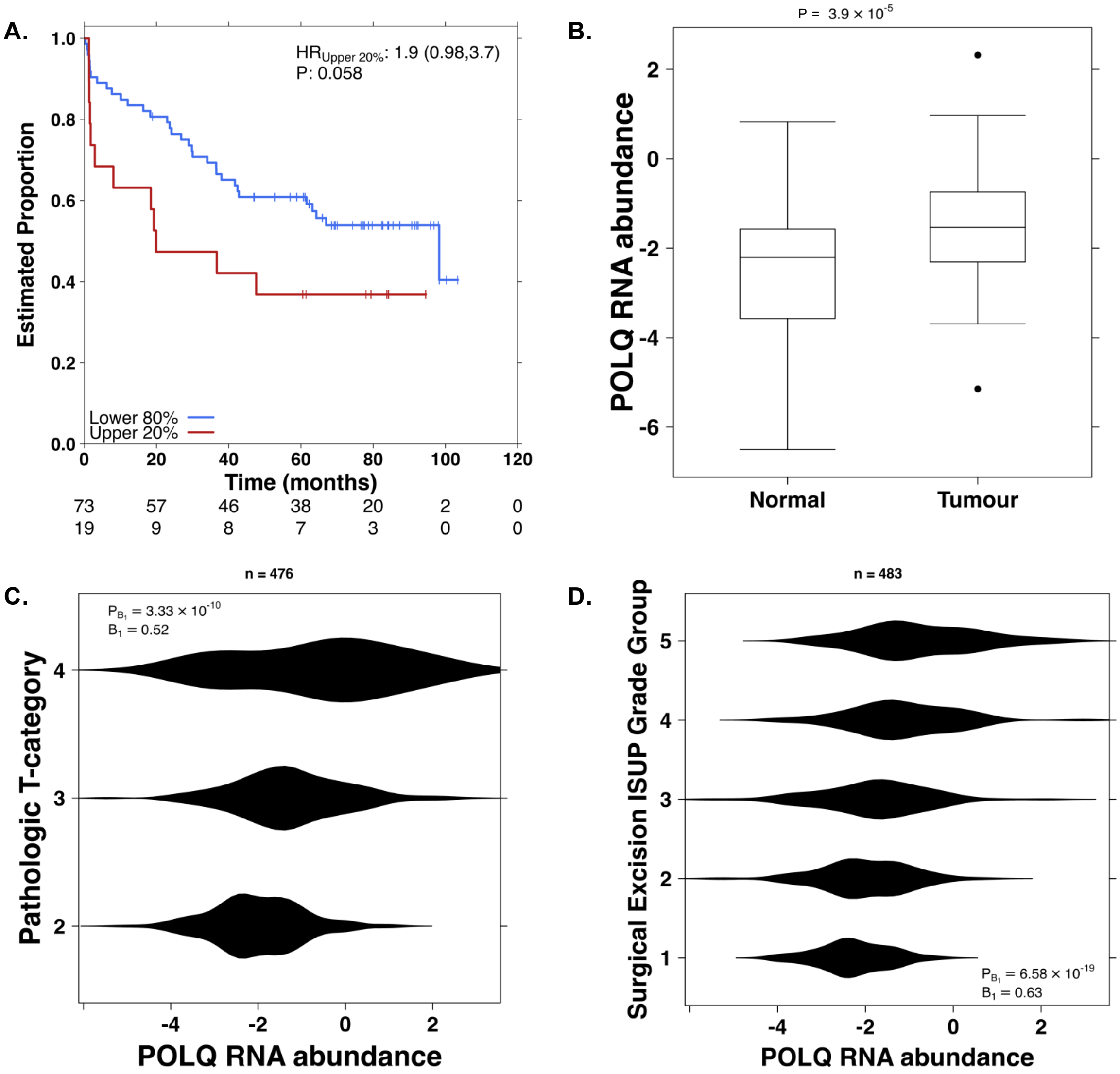

Supplementary Figure 6. Validation of the association of POLQ with prostate cancer aggressiveness. A. High *POLQ* RNA abundance is associated with BCR based on a Cox proportional hazard model. The tested dataset was downloaded from GEO, accession number GSE70770. **B.** RNA abundance of *POLQ* in normal *vs.* tumor samples in 51 TCGA patients with matched normal and tumor samples. P represents the P-value following a paired T-test. **C.** A positive association between *POLQ* RNA abundance and T-categories in TCGA. **D.** A positive association between POLQ RNA abundance and ISUP grade groups in TCGA.
