# Supplementary material for: The Proteogenomics of Prostate Cancer Radioresistance
Source: Cancer Res Commun. 2024 Sep 19;4(9):2463–79. doi: 10.1158/2767-9764.CRC-24-0292 (PMC11411600; doi:10.1158/2767-9764.CRC-24-0292)
Supplement: Supplementary Figure 7 — Association between CNA events in seven candidate genes and BCR, using a log-rank test [file crc-24-0292_supplementary_figure_7_suppsf7.pdf]

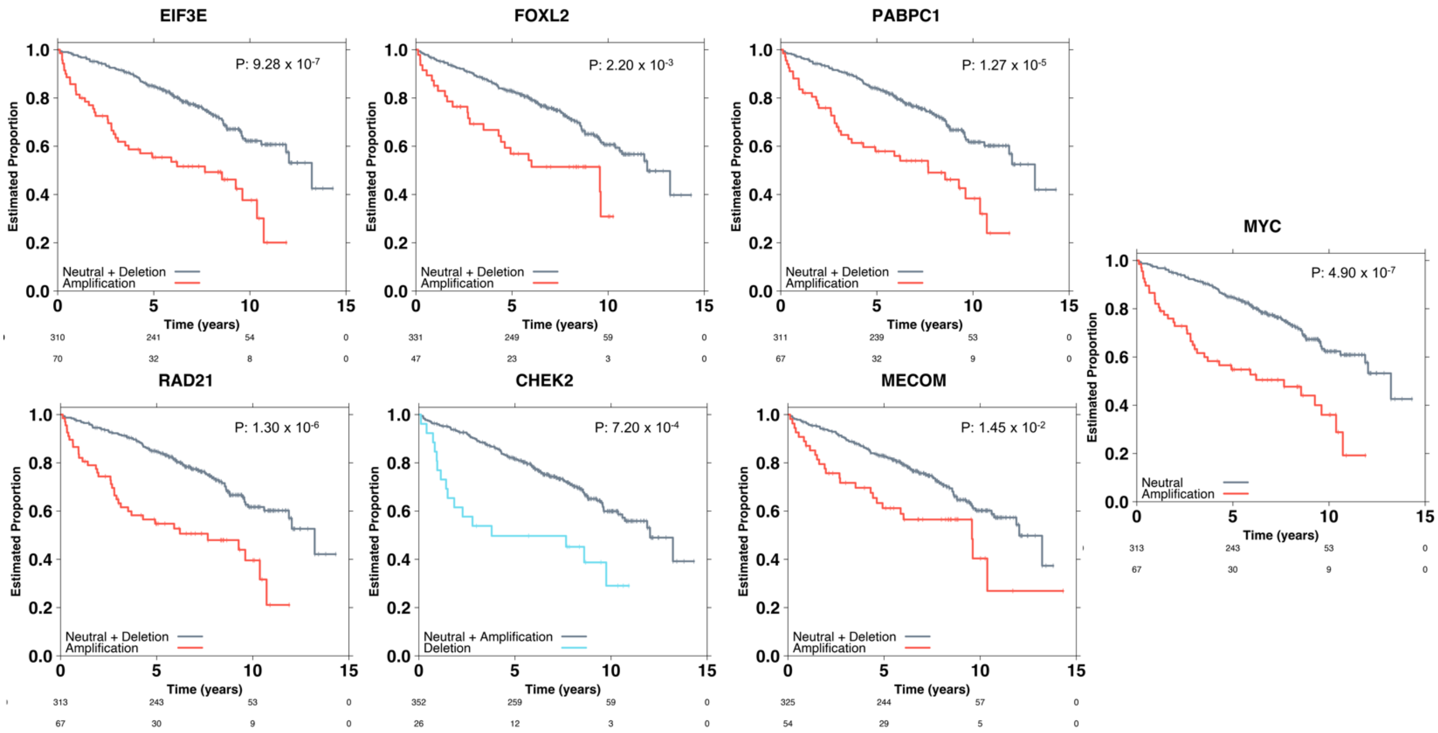

Supplementary Figure 7. Association between CNA events in seven candidate genes and BCR, using a log-rank test. P represents the $P_{adjusted}$ after FDR correction.
