# Supplementary material for: The Proteogenomics of Prostate Cancer Radioresistance
Source: Cancer Res Commun. 2024 Sep 19;4(9):2463–79. doi: 10.1158/2767-9764.CRC-24-0292 (PMC11411600; doi:10.1158/2767-9764.CRC-24-0292)
Supplement: Supplementary Figure 8 — Supporting data for POLQ inhibition effects [file crc-24-0292_supplementary_figure_8_suppsf8.pdf]

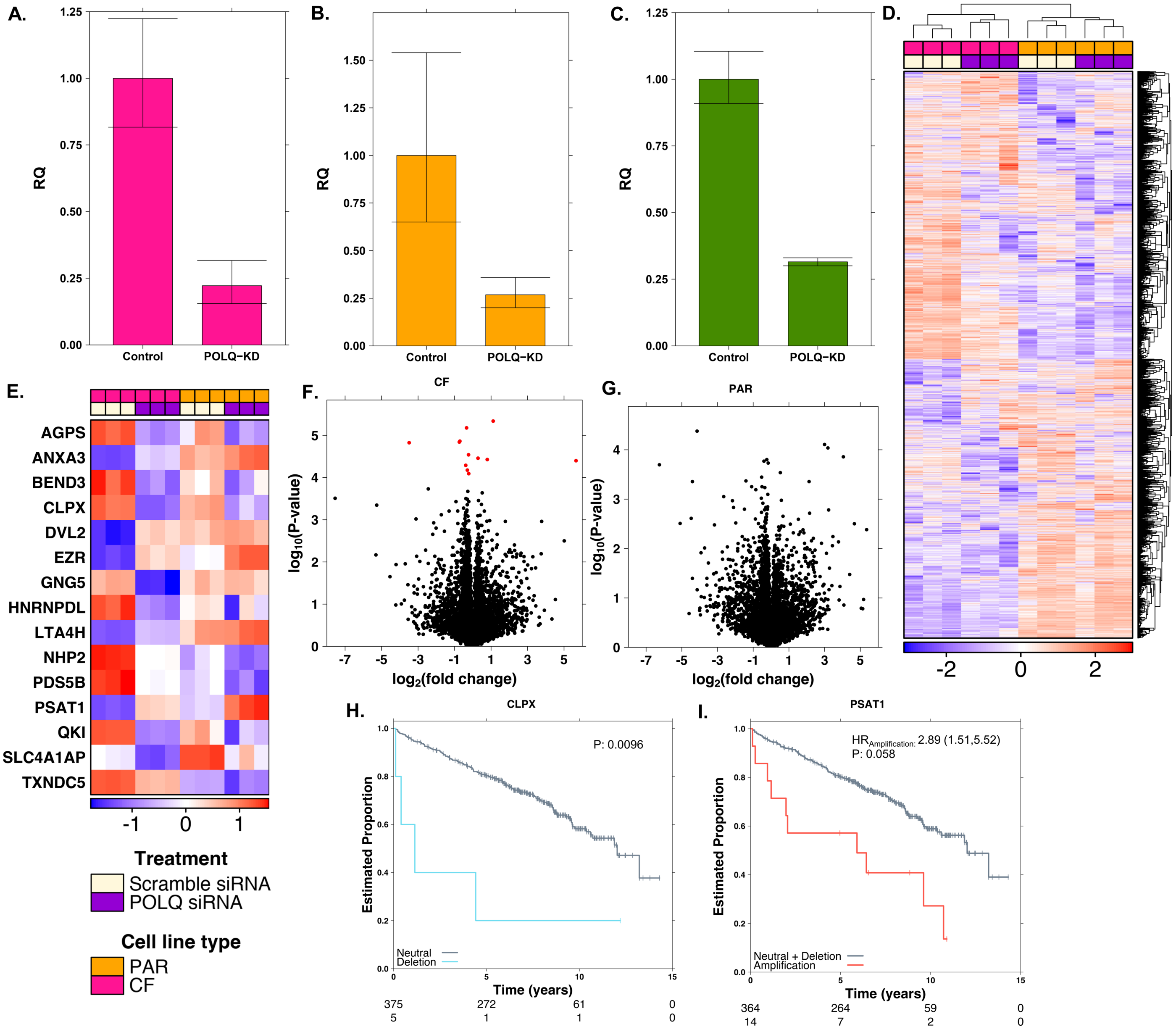

Supplementary Figure 8. Supporting data for *POLQ* inhibition effects. **A-C.** Real-time PCR following *POLQ* siRNA treatments confirmed a successful *POLQ* siRNA depletion, compared to samples treated with scramble siRNA (Control), in CF-resistant cells (**A**), the parental (**B**) and HF-resistant cells (**C**) cells. On the y-axis, RQ is the relative quantification. **D.** The abundances of all detected proteins following *POLQ* knockdown **E.** The abundances of significantly affected proteins in CF-resistant cells, following *POLQ* knockdown. In **D-E**, Red, high intensity; blue, low intensity. For visualization, protein intensities as a function of $\log_2$ were converted to z-scores. **F-G**. Volcano plots for differential protein-abundance analysis, following *POLQ* knockdown in CF-resistant cells **(F)** and the parental cells **(G)**. In **F-G**, red dots represent significant changes in protein abundance (FDR $\leq$ 0.05). **H-I**. Association between BCR and CNA events in the signature genes *PSAT1* and *CLPX*, by fitting a Cox proportional hazard model for *PSAT1* (**H**) and using a Heinze log-rank test for *CLPX* (**I**). P represents the $P_{adjusted}$ after FDR correction.
